# E-Waste in Africa: A Serious Threat to the Health of Children

**DOI:** 10.3390/ijerph18168488

**Published:** 2021-08-11

**Authors:** Tamba S. Lebbie, Omosehin D. Moyebi, Kwadwo Ansong Asante, Julius Fobil, Marie Noel Brune-Drisse, William A. Suk, Peter D. Sly, Julia Gorman, David O. Carpenter

**Affiliations:** 1 Department of Environmental Health Sciences, School of Public Health, University at Albany, Rensselaer, NY 12144, USA; tlebbie@albany.edu (T.S.L.); omoyebi@albany.edu (O.D.M.); 2 CSIR Water Research Institute, P.O. Box AH 38, Achimota, Accra, Ghana; doctorkaasante@gmail.com; 3 Department of Biological, Environmental & Occupational Health Sciences, School of Public Health, University of Ghana, Accra, Ghana; jfobil@gmai.com; 4 Department of Environment, Climate Change and Health, World Organization, 1211 Geneva, Switzerland; brunedrissem@who.int; 5 A World Health Organization Collaborating Center on Children’s Environmental Health, National Institute of Environmental Health Sciences, Research Triangle Park, NC 27709, USA; suk@niehs.nih.gov; 6 A World Health Organization Collaborating Center for Children’s Health and the Environment, Child Health Research Center, The University of Queensland, South Brisbane 4101, Australia; p.sly@uq.edu.au; 7 Graduate School of Humanities and Social Sciences, University of Melbourne, Melbourne 3010, Australia; gormanjwho@gmail.com; 8 A World Health Organization Collaborating Center on Environmental Health, Institute for Health and the Environment, University at Albany, Rensselaer, NY 12144, USA

**Keywords:** e-waste, children, Africa, air pollution, cognitive function, development

## Abstract

Waste electronic and electrical equipment (e-waste) consists of used and discarded electrical and electronic items ranging from refrigerators to cell phones and printed circuit boards. It is frequently moved from developed countries to developing countries where it is dismantled for valuable metals in informal settings, resulting in significant human exposure to toxic substances. E-waste is a major concern in Africa, with large sites in Ghana and Nigeria where imported e-waste is dismantled under unsafe conditions. However, as in many developing countries, used electronic and electrical devices are imported in large quantities because they are in great demand and are less expensive than new ones. Many of these used products are irreparable and are discarded with other solid waste to local landfills. These items are then often scavenged for the purpose of extracting valuable metals by heating and burning, incubating in acids and other methods. These activities pose significant health risks to workers and residents in communities near recycling sites. E-waste burning and dismantling activities are frequently undertaken at e-waste sites, often in or near homes. As a result, children and people living in the surrounding areas are exposed, even if they are not directly involved in the recycling. While toxic substances are dangerous to individuals at any age, children are more vulnerable as they are going through important developmental processes, and some adverse health impacts may have long-term impacts. We review the e-waste situation in Africa with a focus on threats to children’s health.

## 1. Introduction

How to safely dispose of waste products coming from human activity is a global problem. Often in countries without adequate regulation, wastes are simply discarded. Most countries put wastes into landfills, but without adequate control landfills stink, leachate coming from the landfill can contaminate water and methane escaping from degradation of organic material contributes to climate change. In addition, landfills often attract vermin than can cause and spread disease. In many poor countries, people—including children—scavenge landfills for items of value, increasing risk of injury, infection and exposure to dangerous substances. Incineration is an alternative to landfills, but if incinerators are not operated with appropriate controls, they release toxic metals and dioxins that have the potential to cause significant harm to human health [1,2]. 

The harm to the environment is made worse when the discarded material is not readily degradable, as is the case with plastics that now contaminate land, lakes, streams and even the ocean. About eight million metric tons of plastics enter the oceans each year [3]. Hazardous wastes, defined as wastes that are ignitable, corrosive, reactive or toxic, add to the problem and are often intermixed with non-hazardous solid wastes. Hazardous wastes can cause significant harm to human health if not properly contained and disposed, including neuropsychological harm to children [4] and elevations in cancer and birth defects [5].

The term electronic and electrical waste (e-waste) refers to “electrical or electronic equipment, which is waste, including all components, subassemblies, and consumables, which are part of the equipment at the time the equipment becomes waste [6]”. E-waste includes large, discarded appliances, such as refrigerators, air conditioners and washing machines, as well as small personal items, including computers, televisions, mobile phones, and many other devices that are operated by electrical currents or batteries [7,8]. E-waste contains plastics, ceramics, metals, glass, toxic chemicals, such as organic flame retardants and polychlorinated biphenyls (PCBs), and various other potentially hazardous compounds [9,10].

In 2019, the world generated an estimated 53.6 million metric tons (Mt) of e-waste, or an average of 7.3 kg per person [11]. Furthermore, only 17.4% of this was officially documented as properly collected and recycled. Africa generated a total of 2.9 Mt of e-waste in 2019, or 2.5 kg per capita, the lowest regional rate in the world. 

The value of the raw materials in global e-waste is estimated to be 57 billion USD, with iron, copper and gold contributing the most [11]. In some African countries, recycling and dismantling electronic devices has become a major source of employment and income. For example, in Ghana in 2010, an estimated 10,000 to 15,000 people were involved in refurbishing old and second-hand computers while another 20,300 to 33,600 were estimated to be working in recycling and e-waste management [12]. More recently this number has been estimated at more than 40,000 [13]. In 2010, an estimated 201,600 people across Ghana, including families and children, were dependent on e-waste recycling and management for support [12]. This number has almost certainly grown over time.

E-waste includes many substances that are dangerous to the health of humans and the environment if released in an unsound manner. E-waste recorded in 2019 contained as much as 50 tons of mercury and 71 kt of brominated flame retardants [11]. The improper dumping and recycling of e-waste in several African countries serves as a major source for the release of harmful substances. These harmful substances can pollute soil, water, air, dust, and food sources [14,15]. Numerous studies have reported contamination of e-waste workers and local residents with toxic metals, dioxins and furans, brominated flame retardants (BFRs), PCBs, polyaromatic hydrocarbons (PAHs), per- and polyfluoroalkyl substances (PFAS), particulate matter and other air pollutants, phthalates and other chemicals in plastics, and the chemical mixtures at these sites [16,17,18,19,20,21,22]. There are other chemicals present in e-waste for which little information is available. Growing research has found associations between e-waste recycling and a range of adverse health effects, including negative birth outcomes, impaired neurological and behavioral development, impaired thyroid function, and increased risk of chronic diseases later in life [23]. 

While there is significant exposure risk to all individuals who are involved in informal e-waste recycling [24] children are particularly vulnerable to exposure from hazardous chemicals released during informal or unregulated e-waste recycling activities due to their developing organs and immune system, rapid growth, and developmental vulnerabilities. Child labor has been documented at informal e-waste recycling areas across the world. Children as young as five years of age have been observed engaging in e-waste activities in Ghana [12]. Children breathe more air and ingest more food and water relative to their size than adults. As a result, children have higher intakes of pollutants relative to their size than adults. In addition, children’s bodies metabolize and eliminate toxic substances differently compared to adults, making them less able to break down and eliminate some hazardous substances. Children are also closer to the ground, where some toxicants may be at their highest concentration, and are more likely to put their hands, objects, and soil into their mouths, increasing their risk of ingesting contaminants [25,26,27]. Steps taken to protect children will also serve to protect adults. The goal of this review is to assess sources of e-waste, the magnitude of the e-waste problem and e-waste recycling and management practices in Africa and to identify exposures associated with e-waste recycling that pose specific health threats to children and adults. 

## 2. Methods

Peer-reviewed publications within the past 15 years related specifically to e-waste in Africa were assembled based on searches on academic research publication databases including PubMed, Science Direct, and Google Scholar. Publications, governmental and regulatory agency reports, and guidelines, were obtained through formal and informal sources. Keywords used to search the literature were: e-waste in Africa, WEEE in Africa, used electronic equipment in Africa, African e-waste management, e-waste regulations and guidelines in Africa, health effects of e-waste chemicals in Africa, African children’s exposure to e-waste chemicals and impacts, countries exporting e-waste to Africa, and African countries most affected by e-waste. Supplemental Table S1 lists and summarizes the results of original, peer-reviewed research publications that were identified specific to Africa.

## 3. Results

### 3.1. What Chemicals Are Present in E-Waste? 

**Figure 1:**
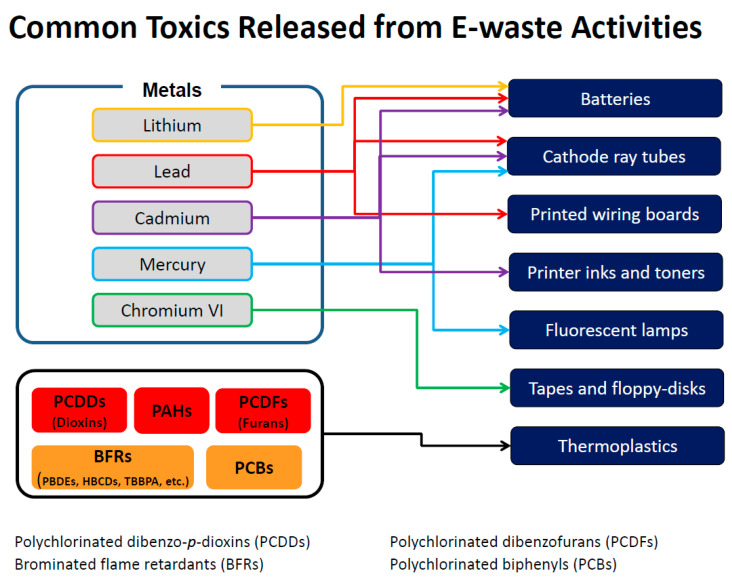
Sources and chemicals released from some specific e-waste sites. Adapted from: Frazzoli, et al. [30].

Tsydenova and Bengtsson [28] reviewed the distribution of toxic chemicals in different items commonly found in e-waste. Their research indicated that common electronic items and their components, such as batteries, switches, relays, and printed circuit boards, may contain antimony, barium, beryllium, cadmium, copper, gold, lead, lithium, mercury, nickel, silver, palladium, and zinc. Items are also known to contain a variety of organic chemicals and rare earth metals, many of which have not been studied for health effects. Plastics may constitute as much as 30% of e-waste by weight [29] and BFRs are added to most plastics to reduce flammability. BFRs are often found in computers and other electronics for the same reason. As many plastics contain chlorine, combustion of plastic results in the formation of both chlorinated and brominated dioxins and furans. Figure 1 shows the sources of some of these chemicals.

Landfills in African countries containing e-waste have shown elevations of many different potentially hazardous metals [31]. Soils around an informal e-waste recycling site in Nigeria have displayed elevated levels of copper, lead, zinc, manganese, nickel, antimony, chromium, cadmium [32]. Alabi et al. [33] compared levels of metals in soils and plants at e-waste sites in China and Nigeria and found that both sites had significant elevations of lead, copper, chromium, nickel, cadmium, and manganese relative to Dutch and Chinese standards. Several reports have investigated blood concentrations of metals in the blood of residents living near e-waste recycling sites as compared to control areas. Li et al. [34] reported that residents living near Taizhou, when studied two years after the site was closed, had elevated levels of chromium, arsenic, cobalt, nickel, silver, tin, mercury, lanthanum, and cerium as compared to residents at a control site. In Ghana, workers at an e-waste recycling site displayed significantly higher concentrations of blood lead, cadmium, chromium, and urinary nickel when compared to non-e-waste workers [35]. Many of these metals are toxic, especially to children. 

### 3.2. How Does E-Waste Pollute the Environment?

E-waste is recognized as a resource as it contains valuable materials [18]. However, in developing countries many e-waste recyclers use primitive methods, such as mechanical shredding, manual dismantling and sorting and open burning, to isolate these valuable materials [16,36]. Plastics are burned, often at low temperatures, to dispose of computer casings and to retrieve metals from electronic chips and other components leading to the formation of dioxins [37]. Old tires may be burned to generate the heat to melt wires and incineration is used to extract valuable materials [38]. Since there are often inadequate stack emission controls, incineration can also release harmful heavy metals into the environment [37]. Strong acids are used to extract metals from printed circuit boards [39]. 

These methods result in severe air pollution containing many toxic substances around e-waste recycling areas. Particulates and other air pollutants are inhaled by workers and nearby residents. In addition, chemicals escape and may contaminate dust, soil, and water in communities around dump sites leading to additional routes of exposure, such as dermal contact and consumption of contaminated food and water [40]. E-waste contains as many as 1000 different harmful substances that have been identified as either components of e-waste or involved in the processing systems engaged by informal recyclers [41]. Fetuses, infants and children are at particular risk as their bodies are undergoing vital development. Exposure to such toxicants results in the disruption of these development processes and may result in long-term health impacts. Physical injuries, including burns, cuts and scrapes and musculoskeletal injuries, are also a major problem among e-waste workers in African countries, due to lack of safety measures, training, and education [42]. Some of the hazardous substances found in e-waste and their associated health effects are discussed in detail below. As very few health effects studies have been completed at African sites, the findings are supported by additional research completed at other international e-waste sites.

### 3.3. Health Effects Associated with Chemicals Found in E-Waste

**Lead:** Lead is a well-known neurotoxin [43]. Even relatively low lead exposure in children can result in a reduction in total intelligence quotient (IQ) and several behavioral abnormalities, including decrease in attention span and increase in frustration and disruptive behavior. At higher exposures, lead can cause anemia, coma and even death. There is no concentration of lead that does not have adverse effects on neurobehavior [44]. Lead exposure at e-waste recycling sites has been associated with altered physical development, increased bone resorption and childhood temperament abnormalities [45,46]. The chain of activities at e-waste sites and the long duration of exposures to lead are major contributing factors to the elevated blood lead levels in African countries [47]. 

**Manganese:** Manganese is an essential nutrient, but both deficiency and excessive exposure can cause disease. Excessive prenatal manganese levels may result in lower birth weight [48] and adverse effects on child neurodevelopment [49]. Rodriquez-Barranco et al. [50], in a meta-analysis of 17 publications, concluded that a 50% increase in manganese concentrations in hair was associated with a 0.7 IQ deficit in children between 6–13 years of age. Manganese exposure from e-waste has been associated with reduced lung function and elevations in levels of malondialdehyde and superoxide dismutase in children aged 8–13 years [51]. 

**Mercury:** E-waste workers are at risk from exposure to both mercury and methylmercury. Methylmercury is formed when elemental or inorganic mercury is deposited in the environment. Methylmercury is a potent neurotoxicant and the major route of exposure is consumption of contaminated fish. The release of mercury compounds near e-waste sites can result in serious contamination of an important food source. Sensitive populations, such as pregnant women and children, are at high risk of severe health effects of mercury inhalation due to its impact on the renal and central nervous systems [52]. Metal artisans, who are at risk of chronic mercury exposure, have displayed prevalence of cough, chest pain, dyspnea, interstitial pneumonitis, and impaired pulmonary function [53]. High blood mercury levels in children aged 3–6 years were associated with elevated 8-hydroxydeoxyguanosine (8-OHdG), an indication of oxidative DNA damage at an e-waste site [54].

**Nickel:** Nickel is a neurotoxic, immunotoxic, nephrotoxic, and genotoxic agent [55]. Some nickel compounds are known human carcinogens while metallic nickel is listed as a possible human carcinogen [56]. Allergic reactions are common effects of nickel exposure, such as skin rashes, dermatitis, and hand eczema. Inhalation of nickel exposure can trigger asthma attacks. Occupational exposure to dust containing nickel has been linked to reduced lung function, nasal sinus, chronic bronchitis, and lung cancer [56]. E-waste workers are vulnerable to these health outcomes. Ni et al. [57] suggested a positive association between nickel and neonatal umbilical cord blood plasma 8-OHdG concentrations. School children residing in an e-waste recycling area have shown significant body accumulation of nickel. Lower forced vital capacity, decrease in catalase activities and significant increase in superoxide dismutase activities and malondialdehyde levels has been associated with nickel exposure in boys aged 8–9 years at an e-waste site [51]. 

**Arsenic:** Arsenic is highly toxic to humans. Studies of children in China have found that elevated arsenic in drinking water is associated with a reduction in childhood IQ [58,59]. Parajuli et al. [60] found that cord blood levels of arsenic were associated with reduced neurodevelopmental indicators in newborns. Liu et al. [61] reported that women exposed to elevated arsenic levels in soil during pregnancy were associated with elevated rates of developmental delays in their children. High urinary arsenic has been associated with decrease in IQ in children between 6–13 years of age [62]. Arsenic is also a potent carcinogen [63] and a major cause of cardiovascular disease [64]. While these diseases may not appear during childhood, exposure during childhood may increase risk of disease later in life. Very little research on arsenic has been conducted at e-waste sites.

**Cadmium:** Cadmium is a known human carcinogen and can have adverse effects on cognitive function. Cadmium has been associated with adverse birth outcomes, reduced cognitive development and IQ and elevated withdrawal, social and attention problems in children [65,66,67,68]. Cadmium exposure in adults is associated with elevated risk of peripheral artery disease [64], but this has not been studied in children. At e-waste sites in China, cadmium exposure has been linked to increased risk of sex-specific adverse birth outcomes and altered mitochondrial respiration [69,70].

**Chromium:** Chromium (VI) is a genotoxic carcinogen, whereas chromium (III) is an essential micronutrient [71]. Children exposed to chromium at e-waste sites have shown lymphocyte DNA damage [72] and reduced weight and chest circumference as compared to unexposed children [73]. Preschool age children from an e-waste site have shown reduced lung function and lower concentrations of hemoglobin in comparison to unexposed children [74]. 

**Polybrominated diphenyl ethers (PBDEs):** PBDEs are a group of BFRs. BFRs can be detected at elevated levels in humans and the environment in areas far away from the points of production, are resistant to degradation and can bioaccumulate [75]. Studies of the health effects of PBDEs on humans have shown significant change in thyroid stimulating hormone in children [76], decrease in full-scale IQ following prenatal exposure [77] and changes in childhood body mass index (BMI) [78] Animal studies indicate that early life exposure to PBDEs promotes obesity later in life [79], and human studies support this conclusion [80]. Elevated concentrations of PBDEs have been found in breastmilk, soil, plants and animals at e-waste sites in China [81,82]. Workers at e-waste sites have shown altered thyroid function associated with elevated serum PBDE levels [82]. Exposure to PBDEs at e-waste sites has also been correlated with reduced head circumference and neonatal BMI, decrease in Apgar1 score, changes to human semen quality and thyroid and endocrine system function [83,84,85,86,87]. 

**Dioxins, furans, PCBs:** Dioxins, furans, and PCBs are known human carcinogens [88,89] Dioxins and furans at e-waste sites are primarily products of combustion. Dioxins, furans and PCBs have been found at elevated levels in soil and air at e-waste sites [90]. There is strong evidence that individuals working at e-waste sites and their children have elevated blood, hair, serum and tissue levels of dioxins, furans, polybrominated biphenyls (PBBs) and PCBs associated with e-waste recycling activities in Ghana [22,91] and China [92,93,94]. PCBs can cause adverse alterations to the nervous system, skin, thyroid and sex steroid hormonal systems, liver, kidney, cardiovascular system, and pancreas [95,96,97]. Children exposed to dioxins, furans and PCBs are at significant risk of respiratory infections and decrease in lung function [98,99]. Exposure to dioxins, furans and PCBs at e-waste sites has been linked to changes in immune system function and significant changes to thyroid and endocrine system function [84,100,101,102]. 

**Perfluoroalkyl substances (PFAS):** There are more than 5000 PFAS widely used as stain resistant and water repellent chemicals on fabrics, household products and as fire-fighting foam [103]. These chemicals have been found in drinking water, food, and indoor and outdoor air [104]. Exposure can result in elevated rates of some cancers [105], altered immune function with a reduced response to immunization [106] and disruption of thyroid hormone function [107]. There is little available research on PFAS compounds at e-waste sites [108] reported that mothers from an e-waste site were exposed to higher levels of perfluorooctanoic acid (PFOA), a common PFAS, and that prenatal exposure was associated with adverse birth outcomes and decreased physical development in children.

**Polycyclic aromatic hydrocarbons (PAHs):** PAHs at e-waste sites are products of incomplete combustion. Workers and residents at e-waste sites encounter PAHs primarily through inhalation and dermal exposure. PAH exposure has been associated with cancers in adults [109,110], DNA damage in newborns [109], respiratory and cardiovascular effects in schoolchildren [111,112] and adverse neuro- and physical development outcomes in children [113,114]. Elevated levels of PAH metabolites have been found in the urine of e-waste workers in Ghana [17]. Cancer risk from inhalation of PAHs near an e-waste recycling area in South China was reported to be 1.6 times that of a control area [115]. Carcinogenic PAH congeners have been positively associated with BMI and child physical growth indicators, such as reduced head circumference and Apgar1 score, at e-waste sites [116,117]. PAHs were observed in preschool children at an e-waste recycling site in China and linked to exacerbated vascular endothelial inflammation [118]. 

**Particulate air pollution:** The joint effects of ambient and household air pollution caused an estimated 543,000 deaths in children under five years and seven million premature deaths across the globe in 2016 [119]. Children in African countries are exposed to a double burden of toxicants from air pollution. While children are exposed to ambient air pollution, including from e-waste activities, they also spend a lot of time at home, indoors, where they may be exposed to toxic pollutants from the incomplete combustion of polluting fuels and technologies used for cooking, heating, and lighting. African countries have some of the highest burdens of disease and disability-adjusted life-years among children due to household air pollution [119]. 

Particulates formed by combustion at e-waste sites will often contain high levels of toxic metals and organic pollutants. Exposure to particulates has been associated with a range of lung and cardiovascular diseases [120,121,122]. An increased association between exposure to particulates and daily hospital admission for respiratory diseases among children five years and below has been reported [123]. Studies have shown that a significant proportional increase in hospital admissions for cardiovascular and respiratory effects can occur even after short-term exposure to particulates [124]. Inhalation of particulates from e-waste burning will aggravate health issues and result in more hospital admissions, especially in children, pregnant women, and the elderly. There is also evidence that early life exposure to fine particulate air pollution is associated with a reduction in cognitive abilities, especially in boys [125]. Outdoor air pollution and the particulate matter within it are classified as carcinogenic to humans [126]. Exposure to particulate matter at e-waste sites has been linked to weakened airway antimicrobial activity in children, increasing vulnerability to respiratory infections [127]. E-waste workers at Agbogbloshie, Ghana have displayed decreases in lung function and heightened risk of respiratory diseases associated with exposure to particulate matter [128]. 

**Other air pollutants:** Air pollution is always a mixture of particulates and gases, and it is often difficult to identify which components are linked to a particular health outcome. The composition of air pollution at e-waste sites depends on items being burned. In Africa, this may be old tires, which when burned will release many different volatile organic compounds (VOCs) either bound or not bound to particulates. A number of VOCs are known human carcinogens [129]. Even VOCs not known to cause cancer may increase the risk of respiratory effects in both children and adults [130,131]. Some VOCs, like benzene, alter hematologic parameters [132]. Inhalation of VOCs at high concentrations can result in a toxic encephalopathy, with effects on memory and behavior [133]. These exposures add to those coming from other indoor and outdoor forms of air pollution.

**Phthalates and other chemicals found in plastics:** Phthalates are a group of chemical compounds that are used as plasticizers and are widely detected in water, soil, and food [134]. There is extensive use of phthalates in many commercial products, and even medical devices [135,136]. Other chemicals, such as bisphenol A (BPA), are also used in plastics. Burning of plastics has been regularly reported as a major issue at e-waste sites. When plastics are burned, phthalates and other chemicals are released into the environment. Both phthalates and BPA are known to be estrogenic chemicals [137]. Studies have found associations between phthalates and many health problems, such as altered semen quality, shortened gestation, reduced anal-genital distance in baby boys, premature breast development in young girls [138], hematological issues [134], and adverse effects on the immune system and neuropsychology [136] Bisphenols at e-waste sites have been linked to abnormal fasting blood glucose levels [139] and oxidative stress [140]. 

**Chemical mixtures:** No one is exposed to only one chemical, and this is especially the case at e-waste recycling sites. Two or more chemicals may show additive, antagonistic, synergistic, or even more complex interactions [50,141]. Goodson et al. [137] found that individual chemicals that are not known to be carcinogens, when present as mixtures, may have all the characteristics required to result in cancer. Pan et al. [142] reported on effects of lead, cadmium, arsenic, and mercury on children’s IQ in an industrialized region of China. While they reported significant effects only for lead exposure, they found that there was a positive interaction between urinary lead and urinary cadmium, but a negative interaction between blood lead and blood mercury. There has been relatively little study of interactions among chemicals at e-waste sites. Exposure to cadmium, chromium and nickel have been associated with increased oxidative DNA damage in neonates [57] and PAH and lead co-exposure has been linked to changes in child growth and development [117]. The issue of exposure to chemical mixtures is particularly important as several chemicals may affect the same or different areas of child development at the same time. The study of chemical mixtures at e-waste sites is difficult, costly and time-consuming to study but needs significantly more attention.

### 3.4. Where Does E-Waste in Africa Come from? 

The growing use and development of electronic and electrical equipment in both developed and developing countries, the affordability of products coupled with lifestyle changes and short product lifespan have contributed to the global growth in e-waste [47,143]. E-waste in Africa comes from three different sources [144]. 

#### 3.4.1. Local Sources 

Electronics manufacturing within Africa is growing. The production, use, recycling, and disposal of these items has also increased dramatically over the past few decades [145]. Local production of e-waste in Africa in 2019 was estimated to be 2.9 Mt, with the largest quantities produced in Egypt (0.585 Mt), Nigeria (0.461 Mt) and South Africa (0.415 Mt) [11]. However, most African countries do not have a formal system for collection and recycling of discarded electronic equipment. In many Africa countries, e-waste is mixed with municipal trash, and landfill scavenging for e-waste items is a major problem [8,146]. There is little documentation on electronic products that are scavenged from landfills.

#### 3.4.2. Import of Used Electronic and Electrical Equipment (UEEE) 

There is growing demand for electronic devices in developed and developing countries. In some low-income countries this has led to increasing import of UEEE from high-income countries. UEEE is more affordable than new electronic equipment. Often, UEEE requires repair, but not all items can be made usable and are discarded, often into landfill where scavengers may collect and dismantle them for valuable metals [147]. As much as 75% of UEEE is shipped legally under the pretense that it is usable or can be repaired. Mureithi and Waema [148] reported that 60% of UEEE given to beneficiaries in Kenya, such as schools, was beyond repair. These items become e-waste. There is also significant import of UEEE into Africa that are mislabeled or hidden with other items, such as sewing machines, motor vehicles and bicycles. In 2015 and 2016, approximately 60,000 tons of UEEE were shipped into Nigeria in this manner [149]. The greatest amounts came from China (24%), followed by the US (20%), Spain (12%) and the UK (9%) [149]. Many of the items are used, irreparable or have outlived their usefulness and contribute significantly to the rapid increase of e-waste in Africa.

**Figure 2:**
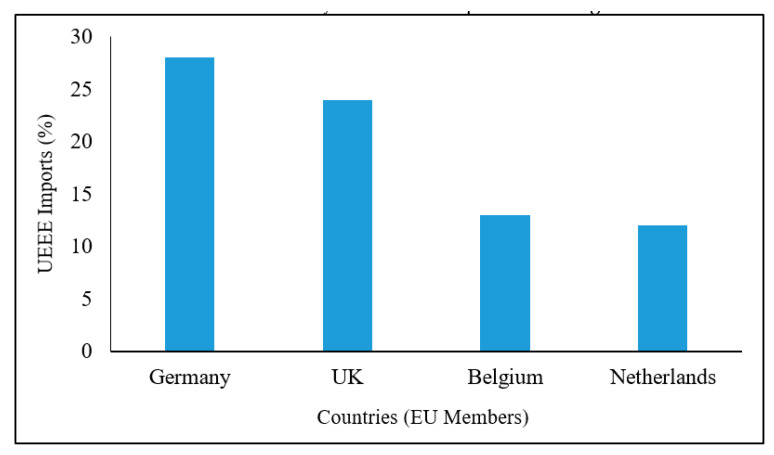
Imported UEEE loaded in vehicles sent to Nigeria from EU countries. Adapted from Balde et al. [150].

Figure 2 shows the percentage of UEEE imported into Nigeria from several European Union (EU) countries within vehicle importation. Many roll-on/roll-off vehicles imported from the EU are loaded with UEEE. Much of it should be declared as e-waste, as many items are beyond repair [150]. It is difficult to obtain accurate documentation of the volume of e-waste arriving in African countries in this way. This contributes significantly to the e-waste problem in Nigeria and other African countries.

**Figure 3:**
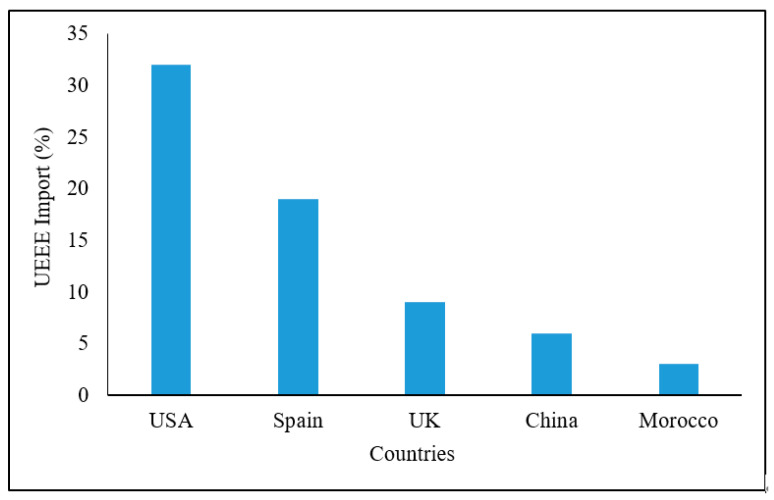
Origins of UEEE imported into the Tin Can Island Port Complex and the Lagos Port Complex, Nigeria in 2015 and 2016 in containers with vehicles. Adapted from Odeyingbo et al. [149].

Figure 3 shows the origins of UEEE inside vehicles imported to two ports in Nigeria. In this case, the largest proportion came from the US, but EU countries, China and Morocco also contributed significantly. 

#### 3.4.3. Export of E-Waste for the Sole Purpose of Disposal

E-waste is exported from high-income countries to low-income countries, where there are often fewer regulations that are poorly enforced, solely for the purpose of disposal. In low-income countries, efforts are made to recover valuable components, such as copper. This is often done in informal settings by untrained workers with little or no protective gear, sometimes near or in homes where children can be exposed to hazardous materials [144]. E-waste also ends up in open dumpsites in African countries, mixed with other wastes, where it is scavenged and recycled by informal workers [145]. 

### 3.5. Which Countries Are Most Affected by E-Waste?

Studies carried out by the European Union and the UN have clearly indicated that African countries, particularly West African countries, have become e-waste dumping sites for developed countries [151]. Low- and middle-income African countries cannot account for the amount of e-waste arriving from other countries due to poor policy frameworks and regulations. In 2019, only 13 African countries had a national e-waste legislation, policy, or regulation [11]. Despite many African countries being signatories to international treaties dealing with the transport of wastes, the lack of regulations and the weak enforcement of those that are in place, encourage illegal e-waste recycling importation and activities [152]. E-waste recycling in African countries occurs under many circumstances. The Agbogbloshie dumpsite in Ghana is the largest e-waste dump in Africa, and one of the largest in the world. It is estimated that about 250,000 tons of sorted electrical and electronic wastes are dumped there annually [13]. About 40,000 people live and work within the environs of this site. In addition to mechanical dismantling of e-waste, much of the e-waste is burned to obtain metals [17]. This results in contamination of air, soil, dust, water, and humans with a wide variety of toxic substances [153,154,155,156,157]. Chicken eggs from Agbogbloshie have recorded the highest level of brominated dioxins and the second- highest level of chlorinated dioxins ever reported [158]. A person eating just one egg would exceed the European Food Safety Authority tolerable daily intake for chlorinated dioxins by 220-fold. While the effects of these contaminants and metals on the local ecology have not been well studied, there is every reason for concern [159,160]. 

Other African countries do not have the same centralized e-waste recycling area. Nigeria does not have any one site as large as Agbogbloshie, but rather has many smaller sites, especially around the major cities of Lagos, Ibadan, and Aba [42]. Many of these sites are markets where UEEE is sold and repaired if needed, and where those that cannot be repaired are dismantled. One of the largest is Alaba International Market. Within the market there is an informal e-waste recycling site where significantly elevated levels of heavy metals in soil have been found [32]. Here, and at other sites, plastic is burned to recover the copper wire, and printed circuit boards are leached with strong acids to recover valuable metals [149]. About 75% of the imported e-waste is directly dumped along roads and in backyards at illegal sites. Babayemi et al. [161] reported that about 16% of e-waste is disposed of through open burning but suggested that this is a considerable underestimation. Sindiku et al. [162] suggested that the burning of the plastics from cathode ray tube casings in Nigeria generates between two and eight tons of PBDEs and furans. 

Three e-waste recycling sites have been identified in Cameroun, but it is likely that there are more. High levels of metals in soil are found at these sites, posing serious risks to residents and e-waste workers [163]. E-waste discarded alongside municipal waste has been recorded in Botswana and South Africa [144,164]. Recyclers in South Africa have reported high exposure to PBDEs and PCBs [165]. Egypt has no national e-waste legislation, policy, or regulation, despite generating the largest amounts of e-waste in Africa [11]. The United Nations Environmental Program [166] summarized the e-waste situation in five different African countries and found that all of them lacked appropriate infrastructure and regulation to safely dispose of e-waste imports. A number of reports have summarized specific e-waste issues in African countries, including Ethiopia [167] Ghana [12] Kenya [168] Malawi [169] Tanzania [170] and Uganda [147,171].

### 3.6. Why Do African Countries Accept E-Waste?

#### 3.6.1. Economic Benefits

E-waste contains materials of significant value, and therefore recycling is an economic opportunity. In 2019, raw materials from e-waste in Africa were worth an estimated USD 3.2 billion [11]. Trade, repair, and recovery of materials from e-waste serve as a source of livelihood for many poor parts of the population. The Agbogbloshie dumpsite in Ghana is estimated to provide livelihoods for approximately 4500 to 6000 workers directly, and up to 1500 people indirectly [40]. Recovery of important raw materials from e-waste has become a business in Ghana and has resulted in global and transboundary trade [40]. Ghana makes an estimated USD 105 to 268 million annually from materials sourced from e-waste and as many as 200,000 people benefit from e-waste recycling activities. Table 1 shows the estimated value of materials from e-waste globally in 2016.

While e-waste does contain valuable materials (Table 1), the cost of recycling safely in developed countries may exceed the economic value of recovered components. However, considering the economic value of gold, plastics, and copper, developing countries will continue to be a dumping site for e-waste by developed countries. Low-income countries often prioritize economic benefits over human and environmental health, thereby exposing their citizens to toxic chemicals. Some high-income countries are guilty of the same misplaced priority.

#### 3.6.2. Lack of Regulation

E-waste legislation is more developed in some parts of the world than others [150]. Supplementary Materials Table S2 shows the breakdown of percentage of the population covered by e-waste legislation in the world and African regions in 2014 and 2017. As of 2017, Western Africa, including Nigeria and Ghana, had the highest regional coverage, but also has significant amounts of imported e-waste. This suggests that despite legislation in place, illegal importation of e-waste continues to frequently occur through poor adherence to, and weak enforcement of, laws. Northern and Southern Africa had no national legislation in 2017 and residents have reported high levels of toxicants in blood samples [165,172].

In 2019, national e-waste legislation covered 71% of the world’s population. However, only 13 African countries had national legislation governing the disposal of e-waste (Table 2) [11]. However, even among those countries with specific legislation, enforcement can be challenging as in many African countries e-waste recycling activities often operate within the informal sector and without significant governmental oversight.

#### 3.6.3. Interventions

The care and management of e-waste in African countries is either poor or non-existent. The management of e-waste in African countries is a major contributing factor to high levels of pollution and hence the adverse effects on humans, animals, and the environment [41]. Few African countries have specific legislation to control or manage e-waste, and where it does exist it is often ineffective (Table 2). A number of regional conventions and localized interventions have attempted to fill in the gaps in national e-waste legislation in African countries. 

The Bamako Convention, *On the Ban of the Import into Africa and the Control of Transboundary Movement and Management of Hazardous Wastes within Africa*, was set up in 1991 to ban the importation of toxic substances from developed countries into African countries. The East Africa Communication Organization (EACO), formed in 2012, has developed working groups to address e-waste issues in its member nations. EACO has held a range of regional conferences and has produced several reports and strategies addressing e-waste management, awareness, and the status of e-waste in member nations. EACO also aims to train national representatives in better e-waste management, facilitating interventions, and the development of more reliable regional and national statistics. At a national level, the e-Waste Association of South Africa (eWASA) was formed in 2008 to establish sustainable and environmentally friendly ways of managing e-waste. Its members include electronics manufacturers, importers, and retailers. eWASA has struggled to affect the import of e-waste in South Africa. One reason for this is that e-waste and its economic benefits have been championed by business-people who may not consider or be aware of short- and long-term health impacts due to exposure of inappropriately recycled e-waste [173]. At the local level, interventions have attempted to target specific health issues with communities that rely on e-waste recycling as a significant source of income. In Agbogbloshie, Pure Earth, has partnered with government agencies and local advocacy groups on a recycling center to improve working conditions through safer recycling of cables and cords. The facility trains recyclers in the use of machines that strip plastics from valuable metals, such as copper and aluminum, and aims to reduce air pollution from cable burning while ensuring that workers do not lose their livelihoods. The Pure Earth facility incorporates feedback from the community and has developed in response to provide more suitable and safe methods of recycling for e-waste workers [174]. In March 2019, the German Government through its Technical Agency for International Development (GIZ) in partnership with the Ghana’s Ministry of Environment, Science, Technology and Innovation (MESTI) established a training workshop to train e-waste workers on cleaner and safer recycling methods; including technical support, to develop a legislation on e-waste, which has now been passed by the Ghanaian Parliament. The key challenges remain not only that stripping machines require longer time and electricity at extra cost to the recyclers to produce the produce quantity of recovered products, but also that no economically viable business model has yet been developed to replace the e-waste burning as a recycling option. The e-waste recyclers have learned that burning cables is a cheaper and faster way of recovering copper than stripping them. Solving the E-waste Problem (StEP) has a number of projects running in African countries. The Person in the Port Project completed in 2017 assessed the quantities, qualities, composition, origins and economic impacts of UEEE imports into two major ports in Nigeria over a two-year period [149]. Other StEP interventions include collaboration between the Ethiopian Government and international partners to develop a national e-waste management strategy and e-waste management pilot projects in West African countries [175]. 

Interventions targeting e-waste are desperately needed in African countries. At international and regional levels, interventions need to target the illegal importation of e-waste, including the better training of inspection officers and better management techniques, without depriving recyclers of their source of livelihood. The International Labor Organization (ILO) has produced strategies on ensuring the safety of e-waste workers that can be adapted to African contexts [176]. At national and local levels, communities that work with e-waste at all levels, from scavengers, recyclers to repairers and re-sellers, must be consulted to design and implement effective and sustainable interventions. E-waste recycling circumstances differ across African countries and communities. Interventions need to reflect the context and specific needs in communities that recycle e-waste. Importantly, interventions at all levels must recognize that e-waste plays an important role in providing income to vulnerable communities and interventions must, therefore, include alternate livelihood options/opportunities for the unskilled e-waste worker to be effective. Interventions must also protect the health of workers, their families and the environment while ensuring that people can maintain their livelihoods.

#### 3.6.4. The Basel Convention and Other Multilateral Environmental Agreements

Awakening environmental awareness, increased environmental regulation and associated disposal costs in developed countries in the 1970s and 1980s led to incidents of uncontrolled transboundary movements of hazardous wastes and, at times, intentional dumping in developing countries, including in the African region. In response, the global community adopted the Basel Convention on the Control of Transboundary Movements of Hazardous Wastes and Their Disposal on 22 March 1989 and entered into force in 1992. The main objective of the Basel Convention is the protection of human health and the environment against the adverse effects resulting from the generation management, transboundary movement, and disposal of hazardous and other wastes.

E-waste can fall under several entries in the annexes to the Basel Convention. In 2019, the “Ban Amendment” to the Basel Convention entered into force, prohibiting the transboundary movement of hazardous wastes controlled under the convention destined for final disposal operations from parties listed in Annex VII (Organization for Economic Co-operation and Development (OECD), the European Commission countries and Liechtenstein) to all other States. The Ban Amendment also prohibits transboundary movement of hazardous wastes controlled by the convention except for those considered or defined as hazardous by the national legislation destined for reuse, recycling, or recovery operations. The entry into force of the Ban Amendment has significant political weight, acting as a flagship of international efforts to ensure that those countries with the capacity to manage their hazardous wastes in an environmentally sound manner take responsibility for them, while still allowing parties wishing to receive wastes required as raw materials for recycling or recovery industries. 

The Basel Convention was followed by other important conventions designed to deal with hazardous wastes, as outlined in Table 3. The Stockholm Convention on Persistent Organic Pollutants is a global treaty to protect human health and the environment from persistent organic pollutants (POPs), which entered into force in May 2004, and addresses some aspects of e-waste management. It initially banned the production and use of 12 specific chemicals, except where specific exemptions apply, and has since added additional hazardous substances to the convention. Several POPs regulated under this convention have been widely used in the manufacture of components of electrical and electronic equipment, namely those made of plastic. Under the Stockholm Convention, articles containing such chemicals must be identified and disposed of in an environmentally sound manner upon becoming waste. In addition, several other chemicals which are regulated by the Stockholm Convention, in particular dioxins and furans, are generated unintentionally through the open burning of e-waste. The Stockholm Convention requires the adoption of several measures to reduce the total release of such chemicals. The Rotterdam Convention on the Prior Informed Consent Procedure for Certain Hazardous Chemicals and Pesticides in International Trade dates from 1998. The goal of this multilateral treaty was to promote transparency in the handling and movements of hazardous chemicals. The Rotterdam Convention promotes open exchange of information and calls on exporters of hazardous chemicals to use proper labeling, include directions on safe handling and inform purchasers of any known restrictions or bans. In 2012, the Secretariats of the Basel and Stockholm Conventions merged with the Rotterdam Secretariat to form a single unit covering some 42 dangerous chemicals. These conventions ban many chemicals that are present in e-waste. Mercury is considered a chemical of major public health concern and as it is present in e-waste items, the Minamata Convention on Mercury is also relevant to reduce its exposure through e-waste recycling activities.

The Bamako Convention is a treaty of the African countries to ban the import into Africa and to control the transboundary movement and management of hazardous and radioactive wastes within Africa. The need for the Bamako Convention was based on the realization that many developed nations were exporting toxic wastes to Africa. This convention was adopted in Bamako, Mali in 1991 and came into effect in 1998 [177]. The major provisions of the above conventions are listed in Table 3.

Ostrava Declaration

- Legal status: Legally binding
- Adoption: 13–15 June 2017
- Entry into force: 15 June 2017
- Parties: 53 Member States of the WHO European Region (as of July 2021)
- Objectives: to improve outdoor and indoor air quality as one of the most important environmental risk factors in the region through actions towards meeting the WHO air quality guideline values in a continuous process of improvement
- Scope: to shape policies and actions on environment and health, support the implementation of effective evidence-based policies and advance actions on environment, health, and well-being in the WHO European Region (as of February 2021)
- Key provisions:
  Improve air quality for all by ensuring access to safe drinking water, sanitation, and hygiene. Minimize the adverse effects of chemicals. preventing, and eliminating the adverse effects of waste management and contaminated sites. Strengthen adaptation to and mitigation of climate change. Build environmentally sustainable health systems

The Basel, Rotterdam, Stockholm, Bamako Conventions, and the Ostrava Declaration are multilateral environmental agreements, which share the common objective of protecting human health and the environment from hazardous chemicals and wastes. Meanwhile, membership of any of the above conventions is not universal and compliance for some countries remains a challenge. For example, while most European countries have signed the Basel Convention, there is evidence that some 352,474 metric tons of e-waste are shipped from EU countries to developing countries each year [178]. Therefore, e-waste management is primarily the responsibility of each government. Too often the economic benefits take preference over the cost to human health. Developed nations must take stronger action to ensure that e-waste produced within their borders does not end up polluting communities in African countries.

## 4. The Way Forward

In 2018, the E-waste Coalition, a group of international organizations working globally and supporting national governments to address the e-waste challenge more effectively and across health and environment, climate and development agendas, signed a letter of intent. The E-waste Coalition has three core functions: *advocacy* including awareness raising and campaigns; *knowledge* and best practice sharing; and the development of a joint *intervention* model for the implementation of e-waste work at the country level [179]. As a member of the E-waste Coalition, the World Health Organization (WHO) is working with international experts and its network of collaborating centers on children’s environmental health and compiling the existing research and knowledge on e-waste and child health, including systematic reviews and regional and global perspectives. The WHO Initiative on E-waste and Child Health also aligns with these goals and has set out aims to produce evidence on the effects of e-waste on child health, build capacity within the health sector to protect children from e-waste exposure and provide relevant monitoring and evaluation of e-waste interventions. As a part of the WHO Initiative, pilot e-waste interventions are being implemented in Latin America, with plans to commence similar pilot projects commencing in Africa [180]. The pilot projects aim to develop a framework for action that can be adapted to interventions at regional and national levels. 

This article proposes that actions can be taken at the local, national, regional and international levels to combat hazardous exposure to e-waste. At the local level, the health sector can play an important role by working with other sectors, such as labor, education, policy and environment, identifying specific needs within e-waste-affected communities, educating local communities on the health risks of exposure to e-waste and communicating methods to reduce exposure where possible, recognizing and monitoring health effects of exposure to e-waste and prescribing appropriate solutions. 

At national, regional and international levels, governments and policy-makers need to ratify appropriate conventions, such as the Bamako and Basel Conventions, that regulate and restrict the transboundary movement of hazardous waste, including e-waste, and implement World Health Assembly Resolution 69.4 on the role of the health sector in the Strategic Approach to International Chemicals Management towards the 2020 goal and beyond. At the regional level, countries need to work together to develop more robust data, build capacity and train those responsible for identifying illegal e-waste exports and to ensure that restrictions on transboundary movement of hazardous waste are enforced. National governments need to develop and implement effective waste management legislation that addresses hazardous waste, such as reducing the use of the most hazardous components in electronic and electrical equipment and encouraging safer and more efficient methods of recycling, develop health-related targets and actions for current and future e-waste policies and conduct research on e-waste and its potential health effects in a variety of contexts and locations, and to evaluate the efficacy of interventions

At national and international levels, the health sector can play an important role by advocating and lobbying policymakers to incorporate strong health protection measures into national waste management policies and new electronics manufacturing initiatives. Across all levels more research is needed to monitor exposure to e-waste, to better understand the health outcomes associated with e-waste and to implement, and provide evaluation, of e-waste interventions. Research is key to providing national governments and international conventions with evidence of the dangers of e-waste exposure, the urgent need for greater regulation and informing effective legislation and controls. At the local level the health sector should be informed and empowered to raise awareness of the risks of e-waste recycling and prescribe appropriate solutions, work with communities at the primary health-care level to educate and engage key agents and implement and monitor the success of interventions that aim to reduce e-waste exposure.

## 5. Conclusions

E-waste is a global problem due to the desire of people to have the latest electronic device, the rapid development of new devices and the short lifecycle of many electronic devices, such as computers and mobile phones. While almost all electronic and electrical items contain valuable materials, especially metals such as copper, the cost associated with extraction of these valuable materials from most devices in a safe manner and then disposing of the remainder often exceeds the value of the materials that are extracted. This has made recycling of electronic equipment questionably cost effective in most developed countries, leading to illegal exportation to and exploitation of less developed countries that do not have stringent safety regulations. At the same time the informal recycling of e-waste has provided necessary income for many people in developing countries. Workers, nearby residents and especially children living in or near the sites of informal e-waste recycling are being exposed to a variety of dangerous chemicals that impact cognitive function and increase the risk of many different diseases, including respiratory diseases and cancer. African countries have tried to address these problems through the Bamako Convention and a number of regional interventions, but lack of national action, legislation and enforcement has resulted in serious harm to e-waste workers, nearby residents and especially to the health of children. Stronger action and awareness are needed at international, regional, national and local levels to ensure that the health of people, especially children, and the environment is protected from hazardous chemicals found in e-waste.

## Figures and Tables

**Table 1:** The potential value of the analyzed raw materials in e-waste in 2019.

Material	Kilotons (kt)	Million USD
Ag	1.2	579
Al	3046	6062
Au	0.2	9481
Bi	0.1	1.3
Co	13	1036
Cu	1808	10,960
Fe	20,466	24,645
Ge	0.01	0.4
In	0.2	17
Ir	0.001	5
Os	0.01	108
Pd	0.1	3532
Pt	0.002	71
Rh	0.01	320
Ru	0.0003	3
Sb	76	644

Global e-waste monitor 2020: quantities, flows, and the circular economy potential [11].

**Table 2:** African countries with e-waste legislation/policy regulations.

Central Africa	Legislation	East Africa	Legislation	North Africa	Legislation	South Africa	Legislation	West Africa	Legislation
Central African Republic	no	Burundi	No	Algeria	no	Angola	No	Benin	no
Cameroon	yes	Comoros	No	Egypt	yes	Botswana	No	Burkina Faso	no
Chad	no	Djibouti	No	Libya	no	Lesotho	No	Cabo Verde	no
Congo	no	Ethiopia	No	Mauritania	no	Madagascar	yes	Cote d’Ivoire	yes
DR Congo	*	Kenya	Yes	Morocco	no	Malawi	No	Gambia	no
Equatorial Guinea	*	Rwanda	Yes	Tunisia	no	Mauritius	No	Ghana	yes
Gabon	no	Seychelles	No			Mozambique	No	Guinea	no
		Somalia	*			Namibia	No	Guinea Bissau	no
		South Sudan	*			South Tome and Principe	yes	Liberia	*
		Sudan	No			South Africa	yes	Mali	no
		Tanzania	Yes			Swaziland	No	Niger	no
		Uganda	Yes			Zambia	yes	Nigeria	Yes
						Zimbabwe	No	Senegal	No
								Sierra Leone	No
								Togo	No

* No information on e-waste legislation/policy regulation. From Forti et al. [11].

**Table 3:** The Basel, Rotterdam, Stockholm, and Bamako Conventions on hazardous chemicals.

**Basel Convention**	**Legal status:** Legally binding **Adoption:** 22 March 1989 **Entry into force:** 10 September 1998 **Number of parties:** 188 (as of February 2021) **Objectives:** To protect human health and the environment against the adverse effects of hazardous and other wastes **Scope:** Hazardous wastes in Annexes I and VIII based on their origin and/or composition and hazardous characteristics listed in Annex III; other wastes in Annex II **Key provisions:** Minimization of the generation of hazardous and other wastes Control system for transboundary movements of hazardous and other wastes based on notification and prior informed consent Environmentally sound management of hazardous and other wastes in relation to transboundary movements
**Rotterdam Convention**	**Legal status:** Legally binding **Adoption:** 10 September 1998 **Entry into force:** 24 February 2004 **Number of parties:** 164 (as of February 2021) **Objectives:** To promote shared responsibility and cooperative efforts among parties in the international trade of certain hazardous chemicals to protect human health and the environment from potential harm and to contribute to their environmentally sound use **Scope:** 52 pesticides, severely hazardous pesticide formulations and industrial chemicals that have been banned or severely restricted for health or environmental reasons by parties and which have been notified by parties for inclusion in the prior informed consent procedure and met the criteria set out in the convention (as of February 2021) **Key provisions:** Prior informed consent procedure based on import responses and export notifications for other banned/severely restricted chemicals Exchange of information on a broad range of potentially hazardous chemicals
**Stockholm Convention**	**Legal status:** Legally binding **Adoption:** 23 May 2001 **Entry into force:** 17 May 2004 **Number of parties:** 184 (as of February 2021) **Objectives:** Protect human health and the environment from POPs **Scope:** 30 POPs (as of February 2021) **Key provisions:** Elimination of POPs, listed in Annex A Restriction of POPs, listed in Annex B Specific exemptions and acceptable purposes for certain POPs Reduction or elimination of unintentionally produced POPs listed in Annex C
**Bamako Convention**	**Legal status:** Legally binding **Adoption:** 30 January 1991 **Entry into force:** 22 April 1998 **Number of parties:** 25 (as of March 2021) **Objectives:** Protect human health and the environment from hazardous wastes in African countries **Scope:** Hazardous wastes in listed Annex 1, or wastes which have the characteristics defined in Annex II as hazardous, or wastes defined by national legislation. **Key provisions:** Prohibit import of hazardous wastes into African countries for any reason Minimize and control transboundary movements of hazardous wastes within the African continent Prohibit ocean and inland water dumping or burning of hazardous wastes Ensure environmentally sound disposal of wastes Promote cleaner production over the pursuit of a permissible emissions approach Establish the precautionary principle

## Data Availability

All data used in this report is publically available as referenced in the bibliography.

## Supplementary Materials

The following are available online at https://www.mdpi.com/article/10.3390/ijerph18168488/s1, Table S1: Peer-reviewed publications dealing with e-waste in Africa, Table S2: Percentage of population covered by e-waste legislation in the world and African regions in 2014 and 2017.
