# Supplementary material for: E-Waste in Africa: A Serious Threat to the Health of Children
Source: Int J Environ Res Public Health. 2021 Aug 11;18(16):8488. doi: 10.3390/ijerph18168488 (PMC8392572; doi:10.3390/ijerph18168488)
Supplement: Supplementary file 1 [file ijerph-18-08488-s001.zip › ijerph-1301634-supplementary.pdf]

Supplemental Table S1: Peer-reviewed publications dealing with e-waste in Africa

S/N	Author/Year	Objective(s)	Methods	Findings/Outcomes
1	Abafe and Martincigh, Environ Sci Pollut Res (2015) 22:14078-14086	To assess the levels of PBDEs and PCBs in e-waste recycling sites in Africa, compare the magnitude of contamination to international data, evaluate the influence of recycling site maintenance on PBDEs and PCBs, and to estimate occupational exposure to PBDEs and PCBs.	Dust samples were taken from two e-waste sites (dismantling/recycling facilities), and one university Information and Communication Technology electronic equipment repair workshop in Durban, South Africa. The collected samples were chemically analyzed by GC-MS for PBDEs and PCBs.	Result shows that PBDEs were detected in all the samples at high levels, especial around e-waste polymers. Daily exposure doses of PBDEs and PCBs suggest that the major pathways of workers' exposure in the workplace are ingestion and dust dermal absorption. So, e-waste recyclers using primitive recycling methods in South Africa are exposed to high doses of bio accumulative tetra- to hexa-BDEs, BDE 209, and PCBs.
2	Ackah, Chemosphere (2019) 235: 908–917	To assess the distribution, contamination and human health risks of Cr, Fe, Cu, Zn, As, Se, Ag, Cd, Sn, Ba and Pb in the top and subsoils of the Agbogbloshie and Ashaiman e-waste recycling sites in Accra, Ghana. To assess non-carcinogenic and carcinogenic risks. To determine the priority metals contributing to the pollution. To explore the relationships among the metals	Topsoil (0 - 30 cm depth) and subsoils (30 - 100 cm depth) were collected from Agbogbloshie and Ashaiman (open burn and dismantling) sites, (n = 180). 39 and 41 topsoils samples were collected from the burn and dismantling areas respectively. 63 and 37 subsoils were collected from the burn and dismantling areas respectively. Control samples were collected at spots where there were no e-waste recycling activities. Topsoils (n = 13) were collected from 4 sampling points in the Ashaiman "Fitter-line" e-waste site.	Maximum concentrations of Cr, Fe, Cu, As, Se, Ag, Cd, Sn, Ba, Pb and Zn in the Agbogbloshie and Ashaiman soils were greater than their concentrations in the control site. Pb showed strong contamination. Agbogbloshie recorded greater Fe, Cu, Zn, Pb, Ba, Sn, Cr, As, Ag, Cd and Se concentrations (average 3.8; 0.7; 0.29; 0.28 wt % and 923; 626; 99; 74; 16; 13; 7 mg/kg respectively as compared to that of Ashaiman. Risk assessment models indicated significant non-carcinogenic health risks in the study areas. As posed carcinogenic risks to children in the burn area subsoil and dismantling area topsoil.
3	Acquah et al., Proceedings of the Human Factors and Ergonomics Society Annual Meeting (2009) 63(1) 938–942	To describe the processes involved in e-waste recycling at Agbogbloshie and discuss some of the associated health and psychosocial challenges.	Direct field observations and in-depth interviews of e-waste workers.	Inappropriate recycling methods, financial constraints, and the high physical demands of e-waste recycling work were associated with adverse musculoskeletal health conditions among the workers.
4	Adusei et al., Ann Glob Health (2020) 86(1) 31	To conduct a spatial assessment and analysis of health conditions associated with different e-waste activities at different	Individuals working in each activity spaces were physically examined and assessed while characterizing and enumerating the scars, lacerations, abrasions, skin	Results indicate that 96.2% of study subjects had cuts, scars, lacerations, and abrasions. Abrasions in 16.3% of the dismantlers. Scars 93.6% of the subjects. Burns was 23.1%. A total of 90.2% of subjects had normal blood

		activity spaces at Agbogbloshie.	condition and cuts. Systolic and diastolic blood pressure values were recorded. Open and close ended questionnaires were administered.	pressure and 9.8% of them were hypertensive.
5	Agyei-Mensah and Oteng-Ababio, Int J Environ Health Res. (2012) 22(6) 500-17	Objective was to explores the perception of health and environmental concerns of workers and residents living close to e-waste recycling sites in Ghana using both qualitative and quantitative research techniques	The methodology includes social constructionist approach, and a logit regression model were adopted to examine the rationale of their claims and concerns which have hitherto been unknown and unexplored	Findings show that workers' environmental and health perceptions were seriously downplayed and do not match those of epidemiological studies, revealing a lack of convergence between lay and expert knowledge. Results further shows a respondent-specificity rationale; the perception of those directly involved in the processes was mainly influenced by economic considerations, while some at best displayed "genuine" ignorance
6	Alabi and Bakare, Toxicol Mech Methods (2017) 27(9) 657-665	Evaluation of the DNA damaging potential of e-waste simulated and raw leachates, and its contaminated underground water using the SOS chromotest and the Ames Salmonella fluctuation test.	Soil samples of raw leachate were collected during rainy season as well as underground water samples within the e-waste dumpsites' vicinity for SOS chromotest and the Ames Salmonella fluctuation test. Physicochemical and heavy metal analysis were carried out using inductively coupled plasma-atomic emission spectrometry.	Finding shows the presence of certain genotoxic and mutagenic substances in the e-waste leachates and contaminated underground water representing direct or indirect risks for all living organisms.
7	Alabi et al., Sci Total Environ (2012) 15(423) 62-72	To compare the level of contamination using soils and plants from e-waste dumping and processing sites in both countries	Levels of PAHs, PCBs, and PBDEs were analyzed using gas chromatography/spectrophotometry and heavy metals using atomic absorption spectrophotometry. DNA damage was assayed in human peripheral blood lymphocytes using an alkaline comet assay.	Findings show that soils and plants were highly contaminated with toxic PAHs, PCBs, PBDEs, and heavy metals in both countries.
8	Asante et al., Sci Total Environ (2012) 424: 63-73	To understand human contamination by multi-trace elements (TEs) in electrical and electronic waste (e-waste) recycling site at Agbogbloshie, Accra in Ghana	38 drinking water samples (boreholes, wells, spring, stream, tap). Spot urine samples randomly obtained from 20 workers (all males) at the e-waste recycling site in Agbogbloshie, Accra, Ghana. Water samples acidified with HNO_3. Concentrations of V, Cr, Mn, Co, Cu, Zn, Rb, Sr, Mo, Ag, Cd, In, Sn, Sb, Cs, Ba, Tl, Pb, and Bi were determined	Concentrations of Fe, Sb, and Pb in urine of e-waste recycling workers were significantly higher than those of reference sites after consideration of interaction by age, indicating that the recycling workers are exposed to these trace elements through the recycling activity. Urinary As concentration was relatively high, although the level in drinking water was quite low. Speciation analysis of Arsenic in human urine revealed

			by ICP-MS. A closed vessel microwave system was employed for the digestion of human urine samples.	arsenobetaine and dimethylarsinic acid.
9	Asante et al., Rev Environ Health (2016) 31(1) 45-8	To explore the different intervention methods used to handle and control E-waste in Ghana	Secondary data from previous studies were used. On the spot observations were also carried out.	The results show that some intervention strategies have been instituted to raise awareness to reduce e-waste burning and to reduce the number children working at the e-waste sites. Also, simple handheld equipments are now available to help in stripping cables as a way of reducing e-waste burning. The results further indicated that more needs to be done to intervene.
10	Asante et al., Current Opinion in Green and Sustainable Chemistry (2019) 18: 109-117	To assess e-waste management systems with a focus on waste disposal, waste reduction, waste reuse, recycling, and recovery in Africa.	Reviewed all available e-waste scholarly articles on material composition and generation, e-waste management and the possible toxic and health effects.	Result shows that e-waste handling and intervention strategies differs from country to country. E-waste is considered a resource base due to its potential for recovering valuable materials and is a source of much-needed income in many low- to middle-income countries. However, E-waste contains toxic substances such as Pb, Cd, Hg, BFRs (such as PBDEs and HBCDD) mostly disposed of by open burning, exposing communities and the environment to carcinogens.
11	Asante, et al., Environ Int (2011) 37(5) 921-8	To elucidation the contamination status by measuring temporal and spatial variations of contaminant profiles of emerging and legacy POPs (PBDEs, HBCDs and PCBs) in human breast milk from Ghana and assess the health risk associated with the intake of these contaminants by infants through breast milk.	Sixty-seven human breast milk samples were collected in 2009 and 2014 from mothers at three locations (Accra, Kumasi, and Primiparous). Demographic information was collected through questionnaire and human milk samples were chemically analyzed for POPs.	PCBs concentrations were higher in all the samples than PBDEs and HBCDs. However, total PBDEs average was higher than what was reported in other studies in Asia and Europe. Industrialized areas have significant sources of persistent contaminants and it is evident that these compounds are still present in Ghana.
12	Babayemi, et al., Environ Sci Pollut Res Int (2015) 22(19) 14502-14	To investigate the material and substance flow of PBDE in the most relevant plastic fractions in Nigeria including the current	Material and substance flow analyses were conducted for the period covering year 2000 to 2010 to study WEEE/EEE using the STAN tool. Sampling and analysis of field activities were carried out as part of the PBDE and	2.4 Mt polymer was imported into Nigeria, 0.8 Mt was still in stocks and 1.6 Mt had reached the end of life. Approximately 1.1 Mt has ended in dumpsites, 0.3 Mt was burned in the open, and 0.2 Mt was estimated to be recycled. Material and substance flow study demonstrates that developing

		knowledge of their fate at the end of life.	BFRs inventory project, which was quantitatively analyzed by GC-MS while samples that tested positive for bromine were analyzed by GC-ECD.	countries in collaboration with appropriate institutions in more industrial countries can develop suitable substance flows as a basis for policy development and waste management.
13	Bortey-Sam et al., Sci Total Environ (2014) 496: 471-478	To determine the concentrations of 22 PAHs including USEPA priority pollutants in surface soil samples from the Kumasi Metropolis. To identify the possible sources of PAHs in surface soils. To develop distribution maps of PAHs throughout the city using and to evaluate the extent of pollution and toxic potential of PAHs in soils	Soil samples were randomly collected from 36 communities in the Kumasi municipality and the pristine site (Kwame Nkrumah University) for comparison. A total of 129 soil samples (0–10 cm top layer) were. The soil samples obtained were stored at − 20 °C	Mean concentration of total PAHs ranged from 14.78 at Ahinsan to 2084 ng/g dry weight at Adum with an average of 442.5 ± 527.2 ng/g dry weight. Results showed that PAHs in surface soil samples from the study area were mainly from fuel combustion. Carcinogenic potency of PAH load from the city centre was approximately 150 times higher as compared to a pristine site and the Kwame Nkrumah University of BaP, a human carcinogen, contributed 70% of the total PAHs toxicity level from the city centre of Kumasi.
14	Burns et al., Int J Environ Res Public Health (2016) 13(1) 140	To characterize the noise levels experienced by e-waste workers at the Agbogbloshie e-waste recycling site. To examine the association between occupational noise exposures and heart rates among these workers over a short period (one day).<br>To evaluate the potential influence of work activities and perceived stress on the observed relationship between occupational noise exposures and heart rates over a short period	Dataset collected as part of a cross sectional study conducted on e-waste workers at Agbogbloshie. Interviewed 57 workers and continuously monitored their individual noise exposures and heart rates for up to 24 hours.	Over 40% of workers had noise exposures that exceeded recommended occupational (85 dBA) and community (70 dBA) noise exposure limits, and self-reported hearing difficulties were common. Moderate to high levels of perceived stress, variety of symptoms that could indicate cardiovascular disease. A mixed effects linear regression model indicated that a 1 dB increase in noise exposure was associated with a 0.17 increase in heart rate (*p*-value = 0.01) even after controlling for work activities, age, smoking, perceived stress, and unfavorable physical working conditions. Occupational and non-occupational noise exposure is associated with elevations in average heart rate, which can lead cardiovascular damage.
15	Burns et al., J Occup Med Toxicol (2019) 14:1	To evaluate injury experience, noise exposures, and stress risk factors among e-waste workers at the large recycling site in the Agbogbloshie market, Accra, Ghana.	Participants' survey addressing their work, health status, stress, exposures to several occupational hazards (including noise), use of personal protective equipment at work, and injury experience. Subset of participants completed personal noise dosimetry	High exposures to noise (43.5% of workers), moderate to high levels of stress (mean PSS score 25 out of 40 possible points). Spearman correlation coefficients between noise and heart rate-highly significant: ρ 0.46 (p < 0.001).<br>1 dB increase in noise exposure associated with a 0.17 increase in heart rate (p: 0.01).

			measurements. Poisson regression was used to evaluate the association between the number of injuries experienced by participants and various factors evaluated in the survey.	
16	Cao et al., Chemosphere (2020) 240	To determine the relative bioavailability of metal(loid)s in soil.	The physiologically based extraction test (PBET) was applied to investigate the bioaccessibility of the metal(loid)s Cu, As, Cd, Sb, and Pb in both coarse and fine soil samples (n = 10) from e-waste open burning sites at Agbogbloshie in Accra, Ghana, was assessed using an in vitro assay, the physiologically based extraction test. A bioaccessibility-corrected HHRA was then conducted to estimate the potential health risks to local inhabitants	The in vitro results (%) varied greatly among the different metal(loid)s (Cu: 1.3–60, As: 1.3–40, Cd: 4.2–67, Sb: 0.7–85, Pb: 4.1–57), and showed marked variance between the gastric phase and small intestinal phase. The particle sizes of soil samples and chemical forms of metal(loid)s also influenced bioaccessibility values. Using these bioaccessibility values, both the hazard index and carcinogenic risk were calculated. The hazard index was above the threshold value (>1) for 5/10 samples, indicating a potential health risk to local inhabitants.
17	Caravanos et al., Journal of Health and Pollution (2011) 1 (1) 16–25	To better understand the multitude of chemical releases at recycling sites	Environmental (ambient) air samples and worker breathing zone samples were taken for selected metals. Surface soil samples were collected throughout the site and analyzed for lead (Pb).	Results of personal air samples collected from workers and the environment revealed there were elevated levels of aluminum, copper, iron, lead, and zinc. More than half of the soil samples were above the US Environmental Protection Agency standard for lead in soil. This further shows that the Agbogbloshie e-waste recycling/disposal site in Accra, Ghana has an extensive lead contamination in both ambient air and topsoil. Given the urban nature of this site as well as the large adjacent food distribution market, the potential for human health impact is substantial both to workers and residents.
18	Chama et al., Journal of Science and Technology (2014) 34(1) 1	To ascertain the level of trace metal contamination in the Odaw River	15 sediment samples were collected from five different locations were analyzed for their trace metals. The locations were chosen to represent areas near to heavy e-waste activities and areas with no apparent e-waste activities, and analysis carried out using Atomic Absorption Spectrophotometry.	Mean concentrations of trace metals (Cu, Cd, Pb, Fe, Cr and Ni) were highest at locations near burning and dumping sites. This is due to e-waste activities and the configuration of the river. Apart from Cu and Cd, the rest of the metals were below the recommended Canadian interim sediment quality guideline (ISQG), none above the Probable Effect Level (PEL). Levels of trace metal contamination were below the

				concentration at which frequent adverse effects are expected to occur. E-waste recycling activities along Odaw River contribute to the contamination of the river.
19	Daso et al., Environ Sci Pollut Res Int (2016) 23(11) 10883-10894	To comprehensively characterize the soils within the Agbogbloshie e-waste dismantling facility for PAHs and to identify the possible emission sources of these pollutant. To evaluate the potential carcinogenic risks associated with exposure to the dumpsite soil by unprotected workers	Isolation with ultrasonic-assisted extraction technique was used. The concentrations of PAHs were determined by gas chromatography mass spectrometry (GC-MS). Loss-on-ignition (LOI) method was employed for the determination of total organic carbon (TOC) of the soil samples	Mean Σ18PAHs were 3006, 5627, 3046, 5555, and 7199 ng g^{-1} dry weight for sampling sites A (mosque), B (dismantling site), C (residential house/police station), D (personal computer repairers' shop) and E (e-waste opens burning area) respectively. Prevalence of phenanthrenes, fluoranthene and pyrene was at all sampling sites. The seven carcinogenic PAHs were the major contributors to the BaPeq concentrations accounting between 97.7 and 98.3 %. Cancer risks at the dumpsite were minimal, however prolong exposure to pollutants at the site may result in increased risk to cancer. 18 PAHs, including the 16 USEPA's priority PAHs and two alkyl-substituted 20 naphthalene were determined at the recycling and dismantling sites. For carcinogenicity, PAHs concentrations were generally within the 'safe' benchmark established for the protection of the environment and human health. PAHs sources include charcoal, wood, energy for cooking, use of old tyres, spent oil and plastics to initiate and facilitate the combustion of obsolete e-waste materials.
19	Daso et al., Environ Monit Assess (2017) 189(8) 418	To evaluate the possible influence of dissolved organic carbon on the concentrations of pollutants in landfill leachate	A total of 24 leachate samples were collected from 8 landfill sites at different locations in Pretoria and Johannesburg, Gauteng Province, South Africa. Samples were collected into pre-cleaned 2.5-L amber bottles and were kept cooled with ice. Filtrate was spiked with known amount of the surrogate standards (13C-BDE 77, 139 and 209). The extraction was performed with ENVI-18™ cartridge previously conditioned with 5 mL each of n-hexane, dichloromethane, and methanol and MilliQ water in	PBDEs were prevalent in the analyzed leachate samples. α-HBCDD, β-HBCDD and TBBPA were not detected. Higher brominated PBDE congeners (Br > 6), except for BDE 154 were also not detected. Lower brominated PBDE congeners were frequently detected. There are no on-site landfill leachate treatment facilities for most of the landfill sites. Contamination of nearby water bodies, surface and groundwater sources could be very common. Given the lipophilic properties of the BFRs investigated. DOC only showed weak to moderate positive correlations with some of the target compounds. Landfill leachate is a complex matrix; as a result, other

			this sequence. PBDEs were eluted with 12 mL of n-hexane/dichloromethane (3:1, v/v), while TBBPA and the HBCDD isomers were eluted with 12 mL of dichloromethane/methanol (4:1, v/v).	factors which are not adequately addressed in this study may have influenced the relationships between DOC and the target compounds in this matrix.
20	Daum et al., Int J Environ Res Public Health (2017) 14(2) 135	To present an integrated review of e-waste studies specific to Accra, with particular emphasis on the well-studied e-waste processing site in Agbogbloshie, and synthesizes the existing research base across interdisciplinary themes of human health, environmental health, globalization, trade and informalization, and public policy	Reviewed all available Ghana-related e-waste scholarly literature published available through PUBMED, SCOPUS, and Science Direct. The Ghana-specific e-waste literature dated back to 2002 and included over 40 sources as of 2016. Used grounded theory approach to evaluate the main content of each article and derive core themes that were the basis for synthesizing the literature.	Circuitry in e-waste traverse multiple borders. Valuable fractions of processed scrap exports such as copper, gold, and rare earth metals can subsidize the sound recycling of non-valuable fractions such as plastics, LED screens, and other residuals. Tacit acknowledgment of the linkages between informal e-waste practices and formal global businesses are key to tackling the present e-waste challenge and its local manifestations. Uncovering the occluded functioning as well as the real impacts of e-waste on human lives, health, and communities has been the major contribution of e-waste research to date. Moving scholarship toward devising and assessing more sustainable solutions that are participatory and fair to low-income workers presents one of the greatest challenges of the next decade.
21	Ennaceur et al., Environ Res (2008) 108(1) 86-93	To investigate levels of OCs in breast milk from Tunisian mothers' resident in different geographic regions, determine the influence of various maternal factors in the contamination levels and evaluate the possible potential risk of OC concentrations, particularly on infants.	Human breast milk samples were collected between February 2003 and January 2005 from 237 mothers in 12 different regions in Tunisia and analyzed by gas chromatography.	Result revealed that the Tunisian general population has been exposed to PCBs and OC pesticides because they were detected in all the analyzed samples. DDTs, PCB 153, 180, and 118 were the chief contributors to the total OC burdens in Tunisian breast milk.
22	Feldt et al., Sci Total Environ (2014) 466-467, 369-76	To assess PAH-metabolite levels in the urine of e-waste workers at one of the largest e-waste dumps in Africa.	Socioeconomic data, basic health data and urine samples were collected from 72 exposed and 40 controls. In the urine samples, concentrations of the hydroxylate PAH metabolites (OH-PAH) 1-hydroxyphenanthrene (1-OH-phenanthrene), the sum of 2- and 9-	Median concentration (μg/g creatinine) of PAH metabolites (1-OH-phenanthrene, 2-/9-OH-phenanthrene, 3-OH-phenanthrene, 4-OH-phenanthrene, 1-OH-pyrene) higher in exposed than control: 0.85 vs. 0.55; 0.55 vs. 0.37; 0.99 vs. 0.63; 0.22 vs. 0.11; 1.33 vs. 0.54 (all $p<0.05$. Tobacco smoking was associated with higher urinary concentrations of 1-OH-phenanthrene

			hydroxyphenanthrene (2-/9-OH-phenanthrene), 3-hydroxyphenanthrene (3-OH-phenanthrene), 4-hydroxyphenanthrene (4-OH-phenanthrene) and 1-hydroxypyrene (1-OH-pyrene), as well as cotinine and creatinine, were determined.	(β: 0.31, p: 0.04), 3-OH-phenanthrene (β: 0.36, p: 0.02), 1-OH-pyrene (β: 0.50, p: 0.02), the molar sum of phenanthrenes (β: 0.32, p: 0.03), & the molar sum of phenanthrenes and 1-OH-pyrene (β: 0.40, p: 0.01) Frequent cough, chest pain & vertigo were reported among exposed workers than control (all p<0.05)
23	Fischer et al., Int J Environ Res Public Health (2020) 17(5) 1534	The aim of this study was to comprehensively assess the health consequences associated with informal e-waste recycling.	A questionnaire-based assessment regarding occupational information, medical history, and current symptoms and complaints was carried out with a group of n = 84 e-waste workers and compared to a control cohort of n = 94 bystanders at the e-waste recycling site Agbogbloshie.	E-waste workers suffered significantly more from work-related injuries, back pain, and red itchy eyes in comparison to the control group. In addition, regular drug use was more common in e-waste workers (25% vs. 6.4%). Both groups showed a noticeable high use of pain killers (all workers 79%)
24	Fujimori et al., Environmental pollution (2016) 209: 155–163	To investigate the interplay between metals and bromine with DRCs in soil from e-waste open burning sites from Agbogbloshie in Accra, Ghana.	Samples of soil/ash mixtures were collected from the Agbogbloshie market in August 2010. Chemical identification and quantification for DL-PCBs, PCDD/Fs, PBDD/Fs, and MoB-PCDD/Fs were carried out by gas chromatography/HRMS.	Similarity in pollution pattern suggests that DRCs, Cu and Pb have a common pollution source from open burning of e-waste such as wires/cables and circuit boards. Soil samples were heavily polluted by toxic metals and DRCs.
25	Gioia et al., Environ. Sci. Technol (2011) 45(4) 1349–1355	To investigate air concentrations of polychlorinated biphenyls (PCBs) in ship-based measurements ~400 km off parts of the West African coast.	Twelve-hour high-volume air samples collected on board the RV Polarstern ship during a cruise from Germany to South Africa. Samples were obtained on Cape Verde Island during the same period to monitor airflows from Africa. Finally, passive samplers were deployed in four West African countries (The Gambia, Sierra Leone, Ivory Coast, and Ghana) to try to characterize potential sources on land.	Air masses came predominantly from Cape Verde and on the ship (~ 95%). Shipboard Σ29PCB concentrations off West Africa ranged from 10 to 360 pg m^{-3} and from 6 to 99 pg m^{-3} in Cape Verde; highest land-based concentrations were observed in Ivory Coast and the Gambia (up to 300 pg m^{-3}), lowest was observed in Ghana (9 pg m^{-3}).<br>Highest PCB concentrations in cruise samples and Cape Verde are recorded when air masses are coming directly from the continent. Importation of PCB-containing wastes to Africa is a possible source. A major "ship graveyard" is known to be in the Bay of Nouadhibou, Mauritania, whereas e-waste dumps occur in Senegal and Ghana and probably widely elsewhere in West Africa. The highest levels of PCBs detected in the cruise and Cape Verde samples come partially from Mauritania, Senegal, the Gambia, and Guinea-

				Bissau. This study clearly indicated that there are important sources of PCBs that are not accounted for in the emission inventories for West Africa
26	Gioia et al., Environ. Sci. Technol (2011) 45 (4) 1349–1355	To ascertain the major African sources of these PCBs. The precise sources of the PCB to be established and used to complete the pollutant inventories of African countries. And proffer solutions to safely dispose of the potentially dangerous chemicals.	Related articles were reviewed	Findings show continuing shift in primary emission sources of PCBs from source regions to the countries of the South such as Africa, where PCBs have not been produced and used. This is supported by PCB levels in abiotic and biotic matrices in Africa. Increases of PCBs concentrations during the last 5–10 years, suggesting potential health risks especially for the newborns. The major sources PCBs in Africa as reported include electronics that imported from the countries of the North, shipwreck, and biomass burning. The threat that PCBs pose to the environment and the fragile health of Africans is a real problem and may become overwhelming in view of prevailing poverty, poor resource allocation and management, strive, and lack of vital infrastructures
27	Heacock et al., Rev Environ Health (2018) 33(2) 219-228	To illustrate the range of activities and conditions of e-waste sites and the health hazards associated with them, describe intervention approaches that may be broadly applicable to diverse scenarios, and to discuss the future prevention and intervention strategies.	Used case studies to illustrate the range of activities and conditions at e-waste sites and the health hazards. Related literature search was done to describe intervention approaches applicable to diverse scenarios, and the future prevention and intervention strategies. Also recognizing the difficult realities of the informal e-waste recycling economy.	One of the key challenges of prevention and intervention studies is addressing the disconnect between the long-term risk from exposure to contaminants because of e-waste recycling activities, and the immediate, acute economic needs of the communities involved in these practices. One of the key challenges of prevention and intervention studies is addressing the disconnect between the long-term risk from exposure to contaminants because of e-waste recycling activities, and the immediate, acute economic needs of the communities involved in these practices. Another key challenge is the fact that the e-waste problem has been growing for decades. Even after primary exposure is reduced or mitigated, the legacy of contamination will remain.
28	Henríquez-Hernández, et al., Environment International (2017) 109: 20–28	To explore the possibility that a potential relationship exists between anemia and toxic and emerging minor elements, in addition	Blood samples were taken from sub-Saharan immigrants arrived at the Canary Islands, Spain during the last years of 2010 decade. Blood samples with anemia were 63 while	Findings indicate that Fe, Cr, Cu, Mn, Mo, and Se were significantly higher in the non-anemia group than in the anemia group. However, higher blood levels of toxic, rare earth and minority elements intensively used in the manufacture of consumer electronic

		to essential elements. To also investigate if immigrants from e-waste recycling sub-Saharan countries show higher exposure to e-waste related elements than those from other countries.	control group without anemia were 78 samples. Agilent 7900 ICP-MS was used to determine the whole blood concentration levels of 48 elements.	devices were found in anemia group, which indicates that they are potentially more exposed to these elements' systemic adverse effects.
29	Henriquez-Hernández, et al., Environmental pollution (2017) 230: 817–828.	To obtain glimpse of the baseline levels of inorganic pollution on the inhabitants of an extensive region of the African continent.	Blood samples were taken from 245 African immigrants from 16 African countries who lodged in shelters within the first two months of arrival. Blood levels were analyzed by Agilent 7900 ICP-MS for elements considered as contaminants and essential elements present in human blood at high concentrations.	Results show that 100% of the samples has Al, As, and V with >90% of the samples also had Cr, Hg, and Pb. Eight elements were significantly higher in women than men, and immigrants from Northern Africa were the most contaminated. Also, higher levels of economic development correlate with higher levels of inorganic pollutants measured in the blood of the population.
30	Isimekhai, et al., Environ Sci Pollut Res Int (2017) 24(20) 17206-17219	To establish the distribution of metals in the e-waste recycling site, assess the contamination/pollution due to the recycling activities and evaluate the potential risk posed to the environment because of the recycling activities.	Soil sampling was carried out at Alaba International Market, Lagos, Nigeria in March, and October 2013 during wet and dry seasons. Trace elements' analysis was conducted by inductively coupled plasma optical emission spectrometry.	Samples from dry and wet seasons differ significantly (p<0.001) for Cd, Cu, Mn, Ni, and Zn where total concentration was higher in the dry season. Findings also shows high level of contamination in the study site with some heavy metals such as Cd, Cu, Pb, Sb, and Zn significantly higher than the soil guideline values.
31	Itai et al., Sci Total Environ (2014) 470-471, 707-16	To measure metal(loid) levels in soil/ash mixtures. To assess the geographical distribution of metal(loid)s by focusing on soil color. To constrain the source of rare elements using correlation to the predominant metals. To compare metal(loid) levels in soil/ash mixtures with previous urinary data. To assess risk according to ingestion of the soil/ash mixture.	Ten soil/ash mixtures, E1 to E10 specified were collected from Agbogbloshie market. The soil/ash mixture samples (0–2 cm depth), which have various colors, were collected using a stainless-steel auger. Concentrations of 15 elements in soil/ash mixtures were measured using a P-XRF spectrometer with Ag anode X-ray tube	Burning of e-waste occurred mainly in two locations. The ground color was dark around the burning sites and gradually changed to a lighter color with increasing distance by mixing with the cover soil. The important outcomes: high levels of Al, Cu, Zn, and Pb in the open burning site; enrichment of rare metal(loid)s (Co, As, Sb, In, and Bi) was found in soil/ash mixtures; good correlation of metal(loid) levels and soil color; and high HQ values of metal(loid) levels were found
32	Iwegbue et al., Science of the	The objective of this study was to evaluate	PBDEs and PCBs in the electronic repair workshop	PBDEs concentrations ranged from 14 to 2578 ng g−1 with a prevalence

	Total Environment (2019) 671: 914-927	the concentrations and risks of human exposure to PBDEs and PCBs in dusts from electronic repair workshops in southern Nigeria.	dusts were extracted with a mixture of hexane/acetone by ultra-sonication, and the extracts were purified with a multilayer silica gel/alumina column. The PBDEs and PCBs in the purified extracts were determined by means of gas chromatography-mass spectrometry. Total of 40 dust samples were collected. These included 10 samples from computer/photocopier/printer repair shops, 20 samples from television/radio/stereo/video player repair workshops and 10 samples from telephone/tablet workshops.	of 85 to 95% for BDE-28, BDE-47, BDE-99 and BDE-100. Hazard indices for PBDEs were >1 for exposure to dust from television/radio/stereo repair workshops whereas the total cancer risk values were within the acceptable range of 10^{-6} to 10^{-4}. The Σ28 PCB concentrations ranged from 96.6 to 3949 ng g^{-1}. The PCB compositional patterns indicate that hexa-PCBs are the most prevalent PCB homologues in the electronic repair workshop dusts. The TEQ for the dl-PCBs in the electronic repair workshop dusts ranged from 2.40 to 4.31 g^{-1}. The estimated hazard indices and cancer risk values suggest that there is a substantial risk (carcinogenic and non-carcinogenic) associated with human exposure to PCBs in dusts from these electronic repair workshops
33	Kaifie et al., Sci Total Environ (2020) 25(723) 138073	The aim of our study was to assess the occupational PCB exposure in e-waste workers in relation to their specific recycling task (e.g., dismantling, burning)	88 e-waste workers and 196 control subjects were included in this study. All plasma participant's samples were evaluated for the PCB congeners PCB 28, 52, 101, 138, 153, 180 and sum of NDL-indicator congeners by human biomonitoring.	A significant difference could be detected for the lower chlorinated PCB congeners (PCB 28, 52, and 101) for e-waste workers in comparison to the control group. Analysing specific recycling tasks, workers who dismantle and those who burn e-waste showed the highest plasma levels of PCB 28 and 52. In conclusion, e-waste workers showed occupational related elevated PCB levels. Although those levels did not exceed the BAT value, workers were contaminated with PCBs during their task.
34	Kyere et al., Environ Health Toxicol (2017) 32: e2017018.	To assess the risk posed to the environment by heavy metals using numerical sediment quality guidelines that represent the thresholds of chemical concentrations associated with the presence or absence of biological effects on communities and ecological risk factors. To examine the spatial extent of the potential ecological risk.	Grid based sampling procedure with 100 m intervals was used. 132 samples were collected from the AEPS. Collected samples were air dried and sieved using a 100 μm mesh and pulverized into a 2.5 cm using a 10- ton hydraulic press. Analysis of the heavy metal concentrations was done using an X-ray fluorescence (XRF) spectrometer at a maximum power of 3000 W (60 kV and 50 mA). The pelleted samples were placed in a disk and then placed on the	Concentrations exceeded the regulatory limits of both Dutch and Canadian soil quality and guidance values. Ecological risk posed by the heavy metals extended beyond the main burning and dismantling sites of the informal recyclers to the school, residential, recreational, clinic farm and worship areas. The heavy metals Cr, Cu, Pb and Zn had normal distribution, spatial variability, and spatial autocorrelation. Organisms in the soil and plants or water bodies in the areas of the clinics, school, parts of the market area, and some parts of the CAs face no ecological risks. Heavy metal contamination presents an important challenge for the AEPS

			excitation source of the XRF for a 10-minute irradiation using a silicon (Si) lithium detector with a resolution of 16 V with manganese and Kα peak used throughout the procedure.	and its surrounding environments (comprising the soil, air, surface and ground water) and consequent implications for terrestrial and aquatic species.
35	Laskaris et al., Annals of Work Exposures and Health (2019) 63(8)	This study aims to address the gaps in exposure assessment in informal settings by using wearable cameras and personal exposure monitoring equipment to generate task-specific exposures	142 workers at the Agbogbloshie e-waste site in Accra, Ghana, wore sampling backpacks equipped with wearable cameras and real-time particle monitors during a total of 171 shifts. Self-reported recall of time-activity (30-min resolution) was collected during the end of shift interviews. Images (N = 35,588) and simultaneously measured PM2.5 were collected each minute and processed to identify activities established through worker interviews, observation, and existing literature. Descriptive statistics were generated for activity types, frequencies, and associated PM2.5 exposures. A kappa statistic measured agreement between self-reported and image-based time-activity data.	Most measured exposures (90%) exceeded the World Health Organization (WHO) 24-h ambient PM2.5 target of 25 μg m−3. The average on-site PM2.5 was 81 μg m−3 (SD: 94). PM2.5 levels were highest during burning, sorting/loading, and dismantling (203, 89, 83 μg m−3, respectively). PM2.5 exposure during long periods of non-work-related activities also exceeded the WHO standard in 88% of measured data.
36	Ledwaba et al., Recycling (2017) 2 (1) 4	The objective of the study was to assess research done on CRT recovery and recycling with the aim of providing a holistic understanding of the current gaps and opportunities that exist within the South African context.	Overview of e-waste management on a global and South African scenario with a specific case for Cathode Ray Tube (CRT) waste management practices in South Africa.	South Africa, the e-waste industry is young and little information is available at a country-level. E-waste ranges 5 % and 8 % of the municipal solid waste in South Africa and these volumes are expected to increase significantly in the near future. South Africa is still faced with many challenges most of which are related to consumer awareness, collection, recycling processes and waste disposal, amongst others.
37	Linderholm et al., Environment International (2010) 36(7) 675-682	To investigate the levels of human exposure to legacy and emerging POPs in a developing West-African country, Guinea-Bissau.	Serum samples from 33 male HIV negative participants who had participated in repeated health examinations between 1990 and 2007 at 5 time points in an open cohort were analyzed by gas	DDT and its metabolites were the major POP found in all pools of serum. Study reported temporal trends decrease for the legacy POPs with increasing levels of BDE-153 among the study participants.

			chromatography for pesticides and PCBs.	
38	Matovu et al., Sci Total Environ (2019) 692: 1106-1115	To assess the levels and possible determinants of PBDEs in human milk in Uganda, and to estimate infant dietary intakes of PBDEs through breastfeeding.	Fifty samples were collected between March and June 2018 and were extracted by dispersive solid-phase extraction (SPE). Clean-up was performed on an SPE column and analysis was done using gas chromatography–mass spectrometry.	2 PBDE congeners were detected in breast milk of mothers from Uganda. BDE-209 was the most predominant congener (contributed 37.1% to the ∑PBDEs). Fish consumption was associated with higher levels of BDE-47. EDIs were less than US EPA reference doses for BDE-47, -99 and -153. RQs were <1 in 96% samples indicating that the milk was fit for infant consumption.
39	Mmereki, et al., J Air Waste Manag Assoc (2015) 65(1) 11-26	To facilitate the understanding of e-waste management system in Botswana and provide key information and insights capable of contributing to the achievement of the goal of developing e-waste policy, set priorities, and propose approaches for effective collection and treatment of e-waste.	Review of e-waste management studies from developed and developing countries to provide insights on cost-effective policies and regulations on e-waste management as well as disposal methods.	There is no single government regulatory agency supervising e-waste management and no monitoring of inflow and movement of e-waste in Botswana. The failure of government to legislate e-waste regulations is putting pressure on the disposal capacity of the municipal authorities.

40	Moeckel et al., Environment International (2020) 137: 105563	To identify pollutants that are likely to be linked specifically to handling of e-waste. To assess environmental concentrations of PAHs, PBDEs, PCBs, chlorinated paraffins, and selected heavy metals in an international context. To distinguish between pollution caused by e-waste and general waste, and identify e-waste-related processes that determine the pollutant concentrations their risk	Organic contaminants (PAHs, PBDEs, PCBs and CPs) and heavy metals and metalloids (Ag, Cd, Co, Cr, Cu, Hg, Ni, Pb, Sb and Zn) were analyzed in samples from two different waste dumps for comparison: the Agbogbloshie e-waste site in Ghana and the Kingtom domestic dumpsite in Freetown (Sierra Leone). The results were compared using principal component analyses (PCA). 10 soil samples (K-1 to K-10) depth of 10 cm was collected at Kingtom and 15 soil samples (A-1 to A-15) at the Agbogbloshie site. The soil samples were analyzed for total organic carbon.	Concentrations of the 16 USEPA PAHs Σ16PAHs ranged from 130 to 5,200 ng/g in the Kingtom soil samples and from 850 to 10,000 ng/g in those from Agbogbloshie. Total concentrations of all PAHs measured (ΣPAH) were 260 to 14,000 ng/g in Kingtom and 1,300 to 16,000 ng/g in Agbogbloshie samples. Due to waste burning at the waste sites and a wide variety of combustion processes in the nearby major cities of Freetown (ca. 1 056 000 inhabitants in 2015) and Accra (ca. 2 500 000 inhabitants in 2019). with 41–81% vs. 12–44%, the contribution of high molecular weight (HMW, 4- to 7-ring) PAHs to total PAHs is significantly higher in the Agbogbloshie than in the Kingtom samples (p = 10-6). With concentrations ranging from 6.3 to 7,700 ng/g, Agbogbloshie samples showed significantly higher (p = 0.010) total PBDE concentrations than Kingtom samples where 1.2–100 ng/g were found. Sum concentrations of all PCBs analyzed ranged from 0.74 to 43 ng/g in Kingtom, significantly (p = 0.003) lower than in Agbogbloshie (6.5–830 ng/g).
41	Nishimura et al., Environmental Pollution (2017) 225: 252-260	To assess the occurrence, profiles, and toxicity of chlorinated polycyclic aromatic hydrocarbons (Cl-PAHs) and brominated polycyclic aromatic hydrocarbons (Br-PAHs) in e-waste open burning soils (EOBS).	In this study, concentrations of 15 PAHs, 26 Cl-PAHs and 14 Br-PAHs were analyzed in EOBS samples.	We found that e-waste open burning is an important emission source of Cl-PAHs and Br-PAHs as well as PAHs. Concentrations of total Cl-PAHs and Br-PAHs in e-waste open burning soil samples ranged from 21 to 2800 ng/g and from 5.8 to 520 ng/g, respectively.
42	Nkabinde et al., Science of the Total Environment (2018) 622-623, 275-281	To investigate the possible BFRs present, a total of 21 dust samples were collected from surfaces of electronic equipment and office furniture and were analyzed using gas chromatography–mass spectrometry (GC–MS)	X-ray fluorescence (XRF) analyzer was employed to measure elemental bromine contents in office furniture and electronics. A total of 21 dust samples were collected from surfaces of electronic equipment and office furniture and were analysed using gas chromatography–mass spectrometry (GC–MS)	Concentrations of ∑7 BDE-congeners ranged from 50 to 3346 ng ng− 1. Of the ∑7 BDE congeners analyzed, BDE-209, − 183 and − 99 were the most dominant congeners. The concentrations observed ranged from < LOD - 1758, < LOD - 401 and < LOD-543 ng g− 1, for BDE-209, − 183 and − 99, respectively. T-HBCDD and 2-ethyl-1-hexyl-2.3.4.5-tetrabromobenzoate (EH-TBB) were detected in 57 and

				67% of the total dust samples analysed with concentrations ranging from < LOD - 673 and < LOD – 385 ng g− 1, respectively. However, Bis (2-ethylhexyl) tetrabromophthalate (BEH-TEBP) was only detected in 24% of the 21 samples exhibiting a concentration range of < LOD - 63 ng g− 1. The detection frequency of 1, 2 Bis (2.4.6-tribromophenoxy) ethane (BTBPE) was 81% with concentrations of < LOD- 1402 ng g− 1. Alongside the legacy BFRs, NBFRs were the most detected
43	Nnorom and Osibanjo, Waste Manag (2008) 28(8) 1472-9	To review the material flow of second-hand and scrap EEE into Nigeria, the current management practices for such wastes in the country and the environmental and health implications of such low-end management practices.	Review of material composition and generation of WEEE such as personal computers being the second largest component next to CRTs in the stream of e-waste.	Under the Basel convention, significant amounts of electrical and electronic equipment are illegally exported to developing countries. Legislation dealing with e-waste in in developing countries is urgently needed in order to stop the dumping of e-waste in low-income countries.
44	Nrior and Kpormon, Current Journal of Applied Science and Technology (2018) 27(6) 1-11	To analyze and compare the effect of two products of spent phone batteries on Pseudomonas sp. in Marine, brackish and freshwater using standard toxicological bioassay	Freshwater and Marine samples were collected from Gokana L.G.A. and brackish sample was collected from Eagle Island (Rivers state, Nigeria). Spent Nokia and Techno phone batteries were purchased. Toxicity testing carried out by dissolving four (4) grams of the spent phone batteries content into one hundred millilitres (100 ml). This served as a stock solution, from which different concentrations (%); 0, 5, 25, 50 and 75, were made; each was inoculated with one millilitre (1 ml) of the test organism (Pseudomonas sp.) and tested for duration 0,4,8,12, and 24 hours respectively using spread plate techniques. The cultures were incubated at 35°C for 18 to 24 hours. Median lethal concentration (LC50)	Percentage logarithm survival of Pseudomonas sp decreased with increasing exposure time and concentrations. (LC50) of the spent phone batteries ranging from 61.76 to 65.31%. Nokia phone battery in freshwater (65.31%) <Techno phone battery in freshwater (65.14%) <Techno phone battery in marine (64.73%) <Nokia phone battery in brackish (64.53%) <Nokia phone battery in fresh water (64.17%) < Nokia phone battery in marine (62.75%) < Techno phone battery in marine (61.76%). The effect of Techno phone battery in marine is the most toxic (LC50 = 61.76%) having the lowest LC50 while Nokia phone battery in freshwater (LC50= 65.97%) has the lowest toxicity effect. These results show that spent phone batteries if disposed into aquatic environments can inhibits normal biological processes within the aquatic ecosystem.

			was determined using SPSS version 20.	
45	Nti et al., Int J Environ Res Public Health (2020) 17(9) 3042	To determine the association between concentrations of PM (2.5, 2.5–10 and 10 µm) in breathing zone and lung function of informal e-waste workers at Agbogbloshie.	Longitudinal cohort study with three repeated measures, 207 participants. 142 healthy e-waste workers and 65 control participants. Lung function parameters (FVC, FEV1, FEV1/FVC, PEF, and FEF 25-75) and PM (2.5, 2.5–10 and 10 µm). Questionnaires were also handed out. Socio-demographic data, respiratory exposures and lifestyle habits were determined using questionnaires. Random effects models were then used to examine the effects of PM (2.5, 2.5–10 and 10 µm) on lung function	PM (2.5, 2.5–10 and 10 µm) above WHO ambient air standards. Small effect estimates per IQR of PM (2.5, 2.5–10 and 10 µm. 10 µg increase in PM (2.5, 2.5–10 and 10 µm) was associated with decreases in PEF and FEF 25–75 by 13.3% % [β = −3.133; 95% CI: −0.243, −0.022) and 26.6% [β = −0.266; 95% CI: −0.437, 0.094]. E-waste burning and a history of asthma significantly predicted a decrease in PEF by 14.2% [β = −0.142; 95% CI: −0.278, −0.008) and FEV1 by 35.8% [β = −0.358; 95% CI: −0.590, 0.125] among e-waste burners. Conclusions: Direct exposure of e-waste workers to PM predisposes to decline in lung function and risk for small airway diseases such as asthma and COPD
46	Oguntoyinbo, Public Health (2012) 126(5) 441-7	To explore the activities of the informal waste management sector in Nigeria, and barriers to integrating them in an inclusive waste management system.	A literature review was undertaken to evaluate the informal waste management system and formal waste management system in Nigeria and other developing countries with similar settings.	Most of the evaluated studies (97%, n = 33) acknowledged the significant environmental and socio-economic roles played by the informal waste collectors and scavengers in developing countries. The studies identified the following as barriers to inclusive waste management in Nigeria: repressive policy, unhygienic waste collection methods, lack of evidence to support activity, and low quality and quantity of secondary materials.

47	Ohajinwa et al., Injury Prevention (2018) 24(3) 185-192	To assess the prevalence, patterns and factors associated with occupational injuries among e-waste workers in the informal sector in Nigeria.	A multistage sampling method to select 279 respondents from three cities (Ibadan, Lagos, and Aba) in Nigeria. A questionnaire was used to obtain information on sociodemographic, work practices and injury occurrences from the respondents in 2015. The data were analyzed using descriptive statistics and standard logistic regression.	High injury prevalence of 38% and 68% in 1–2 weeks and 6 months preceding the study. Common injuries were cuts (59%). Injuries were mainly caused by sharp objects (77%). The majority (82%) of the injuries occurred on the hands/fingers. Only 18% of the workers use personal protective equipment (PPE) and 51% of those that use PPE got at least an injury in 1–2 weeks and 88% got at least an injury in 6 months preceding the study. The factors associated with injury in 1–2 weeks were job designation and the geographical location, while the factors associated with injury in 6 months were job designation, geographical location, and age.
48	Ohajinwa et al., Int. J. Environ. Res. Public Health (2019) 16(6) 906	To gain insights into health risks (cancer and non-cancer risks) associated with exposure to e-waste chemicals among informal e-waste workers via three main routes: Dermal contact, ingestion, and inhalation.	Cross-sectional study design. Ibadan, Lagos, and Aba in Nigeria. PBDE and metals were measured in the dust and topsoil at e-waste sites (burning, dismantling, and repair sites). Adverse health risks were calculated using the EPA model developed by the Environmental Protection Agency of the United States	E-waste workers prone to non-carcinogenic and carcinogenic health risks. Exposure route in order (Dermal, ingestion and inhalation). High need for more appropriate inclusive e-waste management regulations. E-waste chemicals and the health risks at the e-waste sites increased as e-waste recycling activities increased. Control sites < repair sites < dismantling sites < burning sites. Cumulative health risks via all routes of exposure exceeded the acceptable limits of both non-cancer effects and cancer risk at all e-waste sites.
49	Okine, MPhil Thesis submitted to the University of Ghana (2014).	To analyze the flow of UEEE imports into Ghana, how such imports are handled and managed, and further assess the potential environmental and health challenges associated with the current management practices.	The methodology involved analysis of data on the flow of used computer imports to Ghana, observations, and interviews on UEEE imports handling procedures at the Tema Port. Also, heavy metals analysis of soils from control and e-waste sites, and of urine samples from e-waste workers and control group were conducted.	The results indicated that UEEE /e-waste are either imported from developing countries or from developed countries. However, the larger share of such imports is from the developed regions (Europe and North America). Results also show that effective mechanisms for controlling and managing obsolete or non-functional UEEE import flows in Ghana are currently non-existent. Enforcement officials at the port ports of entry do not have the requisite or adequate logistical, technical, and legal capacity to effectively handle

				such flows. Also, significantly higher Pb, Sb, As, Hg and Zn concentrations were found in soil from the e-waste recycling/disposal sites compared with those of the control site. Furthermore, significantly higher levels of Pb, Cu and Zn and Sb were found in urine of e-waste workers compared with those of the control group. Exposure of e-waste workers to these metals could have adverse environmental and health implications
50	Olukunle et al., Environ Sci Pollut Res Int (2015) 22(3) 2145-54	To analyze leachate samples from selected landfill sites to present a clearer distribution of PBDEs in landfill environment	River and landfill sediment samples were collected from selected rivers and municipal solid waste landfill (MSWL) sites across Gauteng Province in South Africa. Leachate samples (2 L each) were collected using grab method in pre-washed and acetone rinsed 2 L amber bottles. Three samples each (water and sediment) were collected from Alberton, Fourie spruit (meyerton), Clarington, Taaiboschspruit in the lowerklip, Vaal and seven points on Jukskei. The seven sampling sites identified on Jukskei River include Eastgate (Marlboro), Midland (Eastgate), N14 (KNP), Bruma Lake, Eastbank, Kyalami and Buccleuch. Collection of sediment samples was described in	The mean and median concentrations of Σ8 PBDEs from river sediment samples was 2.4 and 0.4 ng g−1, respectively, and a range of 0.8–114 ng g−1. The highest concentration of Σ8 PBDEs (43.6 ng g−1) was observed at Jukskei River. Total PBDE concentrations in landfill sediment and leachate samples ranged from 0.8 to 8.4 ng g−1 and 127–3,702 pg L−1. BDE-209 was predominantly detected in most of the sediment samples. Two of the MSWLs gave the highest concentrations of ∑7 PBDEs (2,678 and 3,702 pg L−1). Correlation values for ∑7 PBDEs versus Co ($r = 0.65$), Cu ($r = 0.52$), Mn ($r = 0.10$), Mg ($r = 0.76$), Ca ($r = 0.66$) and Ni ($r = 0.77$) with a statistical significance ($p < 0.05$) were observed except for Na, Cr, Pb, K, Fe and Zn ($p > 0.05$)
51	Orisakwe et al., Journal Health Pollut (2019) 9 (22) 190610	To review the extent of e-waste exposure in Africa and related impacts on people, animals, and the environment.	Four electronic databases (PubMed, Science Direct, Scopus, and Google Scholar) were searched for publications related to e-waste and human health in Africa. Search terms included ‘e-waste in Africa’, ‘e-waste in developing nations’, ‘public health and e-waste’, ‘environment and e-waste’, and ‘e-waste and health’.	Elevated levels of e-waste pollutants in water, air, soil, dust, fish, vegetable, and human matrices (blood, urine, breast milk) indicate that not only are e-waste workers at risk from exposure to e-waste, but the general population and future generations as well. Headache, cough and chest pain, stomach discomfort, miscarriage, abnormal thyroid and reproductive function, reduction of gonadal hormone, and cancer are common complaints of those involved with the processing of e-waste
52	Otieno and Omwenga, J.	To establish the current trends,	Exploratory study and analysis were performed on	Government should put in place effective management mechanisms

	Emerg. Trends Comput. Inform. Sci (2015) 6(12) 2079-8407	opportunity, and challenges in the management of e-waste in Kenya. To recommend measures to be taken to effectively manage or mitigate the effects of e-waste proliferation in Kenya.	the current e-waste management trends in the Kenyan landscape, identifies challenges and opportunities.	for tracking mass flow of e-waste in and out of the country for their sources and distribution channels' identification. Government will also need to engage in Public-Private-Partnerships to build a robust and sustainable infrastructure capable of handling environmentally friendly e-waste management system and promote the informal e-waste sector.
53	Ouabo et al., J Health Pollut (2019) 9(21) 190310	The objective of this study was to evaluate the levels of heavy metals in Douala, Cameroun, the site of e-waste recycling activities.	The methodology involves soil samples been collected from Makea, Ngodi and New Bell e-waste recycling sites, as well as from a control site. Samples were digested and levels of heavy metals were determined.	Results revealed that concentrations of heavy metals in Makea occurred in the order of lead (Pb) (290±40) > zinc (Zn) (160±30) > chromium (Cr) (130±40) > copper (Cu) (130±20) > nickel (Ni) (56±5.7) > cadmium (Cd) (20±3.0); Pb (310±30) >Zn (150±20) >Cu (80±30) >Cr (70±40) >Ni (50±1.0) >Cd (30±5.0) in Ngodi; and Pb (280±40) >Zn (155±35) >Cu (80±50) >Cr (70±40) >Ni (53±2.0) >Cd (20±10) in New Bell. The levels of metals in all the samples were higher compared to the control site, which was composed of vegetation and far from the e-waste sites, and in some cases, higher than permissible limits or guidelines. The ecological risk index of heavy metals for soil samples in all the e-waste sites indicated a very high risk. Heavy metals concentrations in soil around e-waste recycling sites present serious health risks and further investigations are needed.
54	Parra et al., Environmental Pollution (2019) 259:113732	This study determined the PAHs in soils and sediments from Awotan-Asunle in Southwestern Nigeria, to identify the potential sources, and calculated the toxicity and the possible health risk.<br><br>To determine the concentration of 15 PAHs in soil and sediments at different sites from the Awotan-Asunle dumpsite area in the Southwestern region of Nigeria (one	Soil samples collected (0–20 cm) on the four corners and the center and mixed to form a composite representation of soil at each point and were kept in well-labelled aluminum foil bags point 15 points were randomly selected for soil sampling, around the dumpsite (5 points) and the community (10 points). River sediments were sampled at a depth of 0–10 cm below the water surface. A stainless -steel grab was waded into the river until the desired depth was attained and scooped to collect sediments. The sediments	The composition, concentration, distribution, potential sources, toxicity, and cancer health risk of PAHs in soil and sediment were investigated at Awotan-Asunle community, Southwestern Nigeria. The PAHs with the highest concentrations in soils were those with high molecular weights (InP and Cor) and Phe, and for sediments were those with low molecular weight (Phe, Fla, and Pyr); these species tended to be more conserved in the sediments. The diagnostic ratios suggested that soil PAHs were mainly emitted by pyrogenic sources (waste burning). Also, coronene, a marker for fossil fuel burning was present in significant concentration.

		of the largest dumpsites in Africa)	were mixed into a tray. Sediments were collected at 5 points 50 m intervals.	
55	Schluep et al., Electronics Goes Green, findings from the basel convention e-waste Africa programme (2012) 1-6	To present the findings of national e-waste assessments prepared in Benin, Côte d'Ivoire, Ghana, Liberia, and Nigeria related to the amounts of EEE imports, EEE in use, e-waste generated, as well as environmental and socio-economic impacts of the e-waste sector.	Literature review of country assessment reports on e-waste, volumes of imported new and used EEE, e-waste generated, and current recycling practices.	Study found that Ghana and Nigeria in West Africa are the main import hubs and serves as the major trading route of used EEE into the African continent. Policy measures to improve e-waste management in West Africa should refrain from undifferentiated banning of second-hand imports and refurbishing activities and strive for a cooperative approach that includes market and sector associations.
56	Sindiku et al., Environ Sci Pollut Res Int (2015) 22(19) 14489-501	The objectives of this study were to assess the presence of the POP-PBDE and other BFRs. To develop an inventory for Nigeria of POP-PBDEs in plastic of cathode ray tubes from TV sets and computer monitors. To develop impact factors for CRT plastic in EEE/WEEE from different world regions.	382 plastic samples screened for Br, Cl, P, Pb, Hg and Cd using X-ray fluorescence analysis (X-lab 2000. Gas chromatograph (Trace GC Ultra, Thermo, and Dreieich, Germany) coupled with electron capture detection (ECD, 63Ni, 370 MBq). The GC was equipped with a ZB-5 HT inferno (15 m × 0.25 mm × 0.1 μm, Phenomenex, Aschaffenburg, Germany) as stationary phase. Temperatures of GC split/splitless injector and detector were set at 295 and 320 °C, respectively, and the oven temperature was programmed as follows: 140 °C (1 min), 20 K/min (280 °C), 4 K/min (300 °C), 20 K/min (325 °C). Selected samples were analyzed by high-resolution mass spectrometry for confirmation of the BFR mixtures.	A high proportion of the CRT casings (61 %) contained more than 10,000 ppm Br from BFRs. DecaBDE was the major flame retardant used in TV sets and TBBPA for computer CRTs. Average PBDE levels of c-OctaBDE + DecaBDE were 1.1 % TV and 0.13 % PC CRTs. These are above the Restriction of Hazardous Substances (RoHS) limit. These CRT casings are abundant in Nigeria, as in other African countries, in backyards and waste dumps.
57	Sindiku, et al., Environ Sci Pollut Res (2012) 74: 1320-1323	Screening of POP-PBDE and other (brominated) flame retardants in plastic from TV sets and computer monitors present in Nigeria.	Plastic samples from cathode ray tube plastic casings of 158 TVs and 224 computers know to contain c-OctaBDE taken from eight locations (Ogun and Lagos States) in Nigeria were sampled between January	The amount of c-OctaBDE in TVs was estimated to be 2.7 times higher than factors suggested by the SC POP-PBDE inventory guidance. Major flame retardants in the screened TV CRTs were that were at higher levels with c-OctaBDE are DecaBDE, TBPE, and TBBPA. Also, 3 out of the 224

			and March 2011. X-ray fluorescence analysis was used to quantify bromine, chlorine, cadmium, and lead.	computer samples had c-OctaBDE concentrations ranging from 0.87 - 5.09%.
58	Sindiku, et al., Environ Sci Pollut Res Int (2015) 22(19) 14515-29	To investigate the presence and levels of PBDD/Fs in plastics from waste electrical and electronic equipment, including cathode ray tube casings of computer and televisions collected from various e-waste sites in Nigeria.	Total of 382 plastic samples from cathode ray tube plastic casings of 158 TVs and 224 computers know to contain c-OctaBDE taken from eight locations (Ogun and Lagos States) in Nigeria were sampled between January and March 2011. Gas chromatography high-resolution mass spectrometry was used to analyze the targeted brominated flame retardants (PBDD/Fs and PCDD/F).	PBDD/Fs were present in all 52 analyzed television and computer CRT casings with concentrations ranging from 21 to 350,000 ng/g. Average high concentrations of PBDD/Fs were detected in plastics from CRT casings in Nigerian e-waste. It was also reported that estimated 237,000 t of CRT casings in Nigeria contain between 2 and 8 t of PBDD/DFs.
59	Srigboh et al., Chemosphere (2016) 164:68-74	To characterize exposures to Cu, Fe, Mn, Se, Zn and As, Cd, Co, Hg, Ni, Pb elements in the urine and blood of male workers (n = 58) at Agbogbloshie, as well as females (n = 11) working in activities that serve the site, and to relate these exposures to sociodemographic and occupational characteristics.	69 participants recruited, 58 male e-waste workers, and 11 females who sell food and water at the site.<br>A semi-structured questionnaire for socio-demographics and work history. Urine sample (>30 mL) was collected.<br>5 mL of blood was collected. Stored at 4–8 °C	Blood and urinary elements within biomonitoring reference ranges. Blood Cd was 1.2 μg/L median and Pb was 6.4 μg/dl; 67% above U.S. CDC/NIOSH reference level. Urine As was 38.3 μg/L; 39% above U.S. ATSDR levels. Workers who burned e-waste have the highest biomarker levels.
60	Tetteh et al., Glob Health Promot (2017) 24(2) 35-42	To discuss the potential of Health Impact Assessment (HIA) in addressing the health, environmental, and social impacts of e-waste in sub-Saharan Africa	Draw from environmental policy, environmental communication, global health policy, and health communication to argue that managing e-waste could be framed as ongoing HIA where all the steps of HIA are performed on a rolling basis with input from local communities.	Health Impact Assessment (HIA) can help assess the health, environmental, and social impacts of trade policies and programs related to e-waste to maximize the potential benefits and minimize the negative effects. HIA has the potential to optimize health promotion in developing world. It has the potential to address the e-waste problem in Africa.
61	Tokumaru et al., Arch Environ Contam Toxicol (2017) 73(3) 377-390	The objectives of this study were to determine the contamination levels of trace elements and heavy metals using soil and sediment samples as indicators around the e-waste recycling site, Accra,	Inductively coupled plasma-mass spectrometry (ICP-MS) was used to detect the concentrations of trace elements (Mg, Al, V, Cr, Mn, Fe, Co, Ni, Cu, Zn, Ga, As, Se, Rb, Sr, Y, Mo, Cd, In, Sn, Sb, Cs, Ba, Tl, Pb, and Bi) in soils, sediment, human hair, and foodstuff.	High levels of Cu, Zn, Mo, Cd, In, Sn, Sb, and Pb were observed in soils collected from the e-waste recycling sites. The concentrations of Cu, Mo, Cd, Sb, and Pb in human hair were significantly higher than those collected from the control site ($p < 0.01$).

		Ghana, and try to understand the contamination status of humans by collecting and analyzing trace elements and Pb isotopes in fish and the human hair.		
62	Tue et al., Environ. Sci. Technol. (2019) 53(6) 3010–3017	To investigate the distribution and toxic equivalents (TEQs) of brominated and chlorinated dibenzo-p-dioxins/dibenzofurans (PBDD/Fs and PCDD/Fs) in soils from Agbogbloshie e-waste site (Ghana)	The composition of brominated/chlorinated dibenzofurans (PXDFs) and diphenyl ethers (PBDEs, PCDEs, and PXDEs) was examined using two-dimensional gas chromatography–time-of-flight mass spectrometry to elucidate possible formation pathways of dioxins from e-waste recycling.	Highest concentrations of PCDD/Fs and PBDD/Fs found in the open burning (1.3–380 ng/g dry weight) and dismantling areas (11–1000 ng/g dry weight). PXDFs and PXDEs at hundreds of nanograms per gram. PXDFs were formed mainly from PBDFs through successive Br-to-Cl exchange. Monobromo-PCDFs were also derived from de-novo-generated PCDFs in open burning areas. PBDFs contributed similar or higher TEQs (7.9–5400 pg/g dry weight) compared with PCDD/Fs (6.8–5200 pg/g dry weight), whereas PXDFs were also substantial TEQ contributors in open burning areas.
63	Tue, et al., J Hazard Mater (2016) 302:151-157	To describe the contamination levels of PXDD/Fs, the overall toxic equivalents and total DRC emission by examining the surface soils in Agbogbloshie, the largest e-waste recycling site in Ghana.	Soil samples were collected from e-waste site (e-waste burning areas and non-burning areas) and non-e-waste locations in Accra, Ghana. Chemical analyses were carried-out for PCDD/Fs, PBDD/Fs and DL-PCBs as well as PXDD/Fs using gas chromatography-mass spectrometry.	Concentration levels were higher in the open burning areas but lower in the non-burning areas. Meanwhile, the non-e-waste locations recorded two orders of magnitude lower even than the e-waste non-burning areas. Concentrations of PCDD/F and PBDD/F in soils from open burning areas were among the highest reported in informal e-waste sites. The study confirmed serious DRCs contamination at the Agbogbloshie e-waste-site.
64	Wittsiepe et al., Environ Sci Pollut Res Int (2017) 24(3) 3097-3107	To analyze blood, urine, and hair samples from 75 e-waste workers residing in and/or working on a large e-waste recycling site in Agbogbloshie, Accra, Ghana.	A comparative analysis using the Mann-Whitney U test. A cross-sectional study design, analyzed blood, urine, and hair samples from 75 e-waste workers residing in and/or working on a large e-waste recycling site in Agbogbloshie, Accra, Ghana, and compared the results against those of 40 individuals living in a suburb of Accra without direct exposure to e-waste recycling activities	Significant concentrations of Pb (88.5 vs. 41.0 µg/l, $p < 0.001$), Cd (0.12 vs. 0.10 µg/ gcrea, $p = 0.023$), Cr (0.34 vs. 0.23 µg/gcrea, $p < 0.001$), and Ni (3.18 vs. 2.03 µg/gcrea, $p < 0.001$) in urine of e-waste workers than those of controls. No difference in blood cadmium between the groups (0.51 vs. 0.57 µg/l, $p = 0.215$) or in urine mercury levels (0.18 vs. 0.18 µg/gcrea, $p = 0.820$). Hair mercury levels were higher in the controls than in the e-waste workers (0.43 vs. 0.72, $p < 0.001$).

65	Wittsiepe, et al., Environ Int (2015) 79:65-73	To report the levels of PCDD/Fs and PCB in blood samples of workers from the Agbogbloshie EWRS in Accra, Ghana, and controls from the surrounding area without exposure to e-waste recycling.	A cross-sectional study was conducted at the Agbogbloshie EWRS and compared with the control group recruited from Kwabenya North, a suburb of Accra. PCB analysis was conducted in all patients with sufficient sample volume by gas chromatography high-resolution mass spectrometry.	Exposed individuals have significantly higher PCDF congeners than the non-exposed individuals. Results also shows that workers are directly exposed to PCDD and especially to PCDF as combustion products at the Agbogbloshie EWRS, Accra, Ghana. In addition, relatively high PCB exposure that was found in the control group could be due to consumption of contaminated fish.
66	Yu et al., Glob Health Promot (2017) 24(4) 90-98	To investigate the electronic waste workers' knowledge about the potential health hazards associated with their work as well as the livelihood alternatives that they would prefer if they were given the opportunity.	Cross-sectional study used to gather empirical information on e-waste workers' knowledge about the potential hazards associated with their work and the livelihood alternatives to e-waste recycling with a sample consisting of twenty all-male electronic waste workers at the Agbogbloshie scrap metal yard in Accra, Ghana.	E-waste workers were exposed to a variety of injuries and illnesses. Workers' knowledge of association between health status and work was generally poor. Rather than physical injuries, they did not believe their work played any negative role in their health conditions. They preferred occupations such as farming or professional driving located in the northern region of Ghana to be closer to their families.

Abbreviations:

As- Arsenic, Sb- Antimony, Se- Selenium, Zn- Zinc, Cd- Cadmium, Fe- Iron, Hg- Mercury, Ni- Nickel, Pb-Lead, Br- Bromine, Cr- Chromium, Mn- Manganese, Tl- Thallium.

ATSDR- Agency for Toxic Substances and Disease Registry, **BFR**- Brominated Flame Retardant, **CDC**- Centers for Disease Control, **CRT**- Cathode Ray Tube, **$CuCl_2$**- Copper (II) Chloride, **C-OctaBDE**- commercial Octabromodiphenyl ether, **dBA**- Decibel (unit of measurement of noise level), **DecaBDE**- Decabromodiphenyl Ether, DL-PCBs- Dioxin- like polychlorinated biphenyls, **DNA**- Deoxyribonucleic acid, **DRCs**- dioxin-related compounds, **EEE**- Electrical and Electronic Equipment, **HBCDs**- Hexabromocyclododecane, **HIA**- Health Impact Assessment, **ICP-MS**- Inductively coupled plasma mass spectrometry, **NIOSH**- National Institute for Occupational Safety and Health, **PAHs**- Polycyclic Aromatic Hydrocarbons, **PBDEs**- Polybrominated diphenyl ethers, **PCBs**- Polychlorinated biphenyls, **PCDDs**- Polychlorinated dibenzodioxins, **PCDFs**- Polychlorinated dibenzofurans, **TBBPA**- Tetrabromobisphenol A, **TEQ**- Toxic Equivalents, **WEEE**- Waste Electrical and Electronic Equipment

Table S2: Percentage of population covered by e-waste legislation in the world and African regions in 2014 and 2017.

**Region**	**2014**	**2017**
World	44%	66%
East Africa	10%	31%
Middle Africa	14%	15%
Northern Africa	0%	0%
Southern Africa	0%	0%
Western Africa	49%	53%

Balde et al. (2017)
